# PANoptosis in diabetic retinopathy: immunological insights into mechanisms and translational therapies

**DOI:** 10.3389/fimmu.2026.1668099

**Published:** 2026-06-19

**Authors:** Lingli Ma, Ning Hou, Xiaoyu Zhao, Zimeng Li, Qing Liu, Jianyu Zhao, Qing Wang

**Affiliations:** 1 Department of endocrinology and metabolism, China-Japan Union Hospital of Jilin University, Changchun, China; 2 Department of Cardiac Superficial Musculoskeletal Ultrasound, Songyuan Central Hospital, Songyuan, China

**Keywords:** biomarkers, diabetic retinopathy, PANoptosis, programmed cell death, therapeutic strategies

## Abstract

Diabetic retinopathy (DR), a leading cause of blindness, is driven by hyperglycemia-induced neurovascular damage. Emerging evidence indicates that PANoptosis, an integrated inflammatory programmed cell death modality encompassing apoptosis, pyroptosis, and necroptosis, participates in the progression of diabetic retinal damage; however, the supporting evidence varies substantially across clinical specimens, diabetic animal models, high-glucose cultured retinal cells, and non-DR inflammatory disease models. This review systematically summarizes the latest advances in PANoptosis-associated mechanisms underlying DR pathogenesis, focusing on PANoptosome signaling networks, non-coding RNA-mediated regulation, and immune-metabolic crosstalk. We outline promising candidate biomarkers including PANoptosis-related gene signatures and inflammatory cell death molecules, and critically evaluate multiple translational therapeutic strategies covering small-molecule inhibitors, gene intervention, and nanomedicine delivery. Importantly, we further address context-dependent dual roles of PANoptosis, potential safety risks of non-selective PANoptosis blockade, challenges in blood–retinal barrier penetration, systemic immune side effects, and pathological heterogeneity among DR subtypes and disease stages. Since PANoptosis also fundamentally contributes to host defense and retinal innate immune homeostasis, DR therapeutic strategies should prioritize biomarker-guided, local ocular delivery, time-dependent stage intervention, and cell-type-specific fine tuning, rather than generalized systemic suppression. By reconciling mechanistic progress with unresolved translational bottlenecks, this review proposes that PANoptosis serves as a dynamic and evolving conceptual framework for interpreting inflammatory neurovascular degeneration in DR, instead of an entirely confirmed clinical therapeutic target. We emphasize the urgent need for DR-specific mechanistic verification, longitudinal biomarker cohort studies, and well-designed multicenter clinical trials to advance safe and precise targeted interventions.

## 1 Introduction

Diabetic retinopathy (DR), a leading cause of blindness among working-age populations worldwide, is one of the most common microvascular complications of diabetes, with an incidence of 34.6% among diabetic patients (1). Characterized by progressive retinal neurodegeneration, vascular dysfunction, and neovascularization, the pathogenesis of DR involves a complex interplay of hyperglycemia-induced oxidative stress, inflammation, and aberrant cell death (2, 3). While traditional mechanisms highlight microvascular damage and angiogenesis driven by vascular endothelial growth factor (VEGF), emerging evidence underscores the critical role of programmed cell death (PCD) pathways, particularly PANoptosis—a recently defined inflammatory cell death modality that integrates apoptosis, necroptosis, and pyroptosis (4).

Initial studies recognized PANoptosis as a host defense mechanism against infections, regulated by multi-protein PANoptosomes that coordinate caspase-8, Receptor-interacting serine-threonine kinase 3 (RIPK3), and Nucleotide-binding oligomerization domain-like receptor protein 3 (NLRP3) signaling to release pro-inflammatory cytokines such as Interleukin-1 beta (IL-1β) and danger-associated molecular patterns (DAMPs) (5). In DR, hyperglycemia can aggravate mitochondrial dysfunction through dynamin-related protein 1 (Drp1)-mediated mitochondrial fission, leading to reactive oxygen species (ROS) overproduction and oxidative mitochondrial DNA (mtDNA) stress (6, 7). These events may provide upstream danger signals for PANoptosis-related inflammatory cell death; however, direct evidence confirming complete PANoptosome assembly in specific diabetic retinal cell types remains limited (8, 9). However, although anti-VEGF therapies alleviate vascular leakage, they do not address neurodegeneration, underscoring an unmet need to target PCD pathways that underlie retinal cell loss.

Current research on PANoptosis in DR remains fragmented, with significant gaps in understanding how PANoptosome complexes drive neurovascular injury, the role of non-coding RNAs(ncRNAs) in modulating PANoptosis, and the translational potential of PANoptosis inhibitors (10). For example, although machine learning has identified PANoptosis-related genes as diagnostic biomarkers, their functional validation in DR progression and therapeutic targeting remain underexplored. Additionally, the crosstalk between PANoptosis and immune cell infiltration in sustaining retinal inflammation is poorly characterized.

This review synthesizes recent advances in PANoptosis-related mechanisms in DR, including PANoptosome-associated signaling, ncRNA regulation, metabolic stress, immune-cell crosstalk, biomarkers, and therapeutic strategies. We adopt a balanced and evidence-driven view to avoid one-sided emphasis on the therapeutic value of PANoptosis inhibition. Specifically, we distinguish direct mechanistic evidence from DR clinical and experimental models from indirect inferences derived from other retinal disorders or systemic inflammatory conditions, and interpret PANoptosis as a context-dependent biological process that contributes to retinal neurovascular injury under hyperglycemia, while also participating in host defense, innate immune surveillance, and physiological clearance of damaged retinal cells (8, 11, 12).

Therefore, future therapeutic strategies should focus on biomarker-guided, locally delivered, temporally, controlled, and cell-type-specific modulation of PANoptosis-related pathways rather than broad systemic inhibition. This evidence-based framework clarifies both the research prospects and inherent limitations of PANoptosis, offering a reasonable reference for early diagnosis and precision therapeutic intervention in DR.

## 2 Pathogenic mechanisms of DR

### 2.1 Hyperglycemia-induced oxidative stress

**Figure 1:**
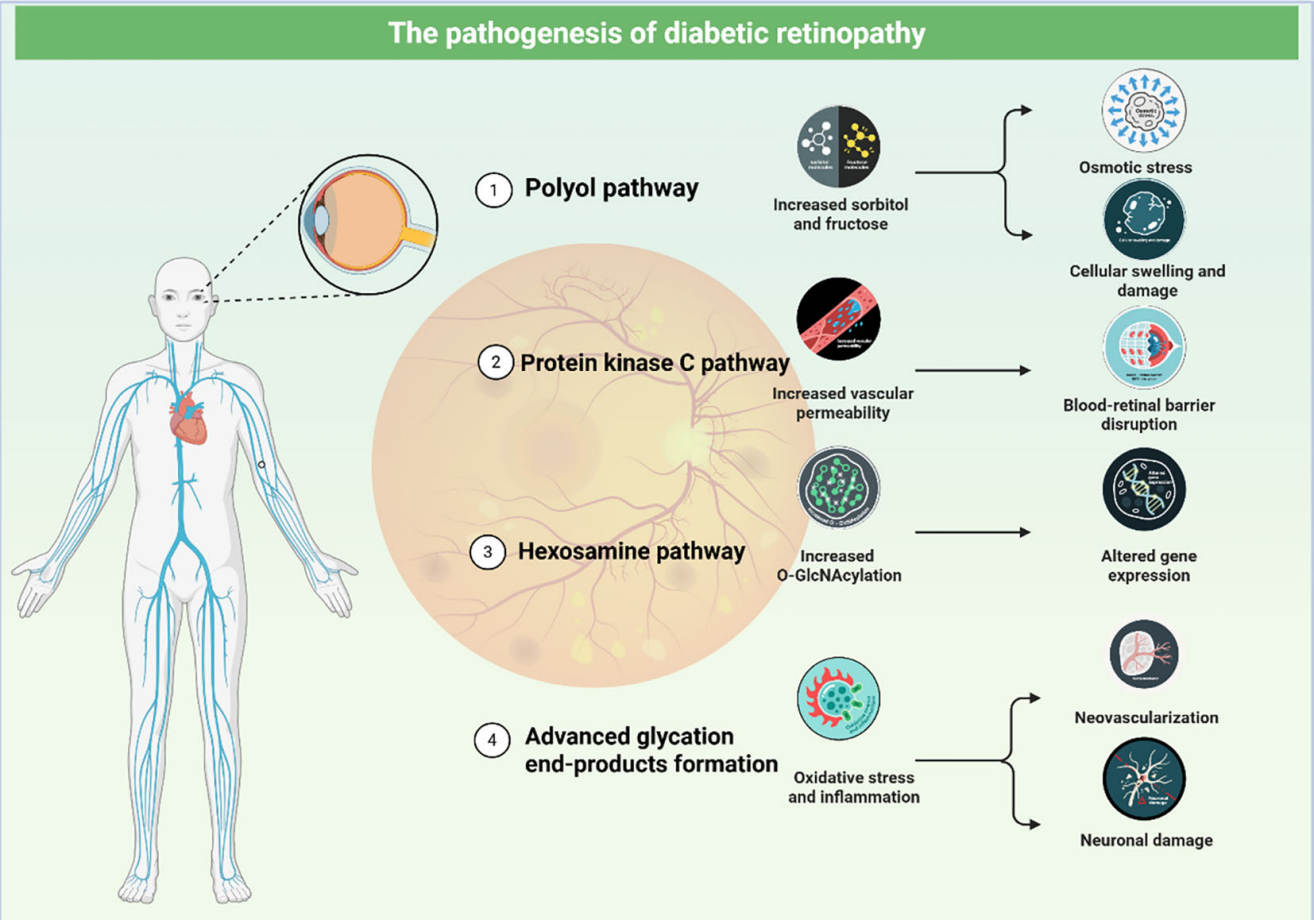
Schematic diagram of hyperglycemia-driven pathways in diabetic retinopathy. This figure depicts the mechanistic network in which hyperglycemia, as the central initiating factor, activates four major biochemical pathways in DR: hyperglycemia promotes the production of sorbitol and fructose through the polyol pathway, inducing osmotic stress that leads to retinal cell swelling and damage; it enhances vascular permeability and disrupts the blood-retinal barrier (BRB) via the protein kinase C pathway; it regulates gene expression through increased O-GlcNAcylation via the hexosamine pathway; and it triggers oxidative stress and inflammation by causing (AGEs) to bind to their receptors, thereby inducing neovascularization and neuronal damage. The detrimental effects of these pathways culminate in retinal cell death, propelling the pathogenesis and progression of DR.

Chronic hyperglycemia is the cornerstone of DR pathogenesis, triggering excessive production of ROS via mitochondrial dysfunction, the polyol pathway, and glucose autoxidation. The retina’s high metabolic demand and its abundance of polyunsaturated fatty acids make it particularly susceptible to oxidative damage (13). For instance, hyperglycemia activates aldose reductase in the polyol pathway, which converts glucose to sorbitol, resulting in osmotic stress and Nicotinamide Adenine Dinucleotide Phosphate Hydrogen (NADPH) depletion (14, 15). This exacerbates the burst of mitochondrial ROS. Accumulated ROS overwhelm antioxidant defenses, leading to lipid peroxidation, protein carbonylation, and DNA damage in retinal neurons, endothelial cells, and pericytes (16). This oxidative insult initiates apoptotic cascades and disrupts cellular bioenergetics, serving as an early driver of retinal degeneration (17) (**Figure 1**).

### 2.2 Inflammation and immune activation

DR is characterized by chronic low-grade inflammation, with pro-inflammatory cytokines such as Tumor Necrosis Factor Alpha (TNF-α), Interleukin-6 (IL-6), and IL-1β, along with immune cell infiltration, serving as key mediators (18, 19). Activated microglia and macrophages secrete TNF-α, which destabilizes retinal vascular endothelial tight junctions, thereby increasing blood-retinal barrier (BRB) permeability and promoting edema (20). IL-1β triggers NLRP3 inflammasome activation, driving pyroptotic cell death and amplifying cytokine release in Müller cells and retinal ganglion cells (RGCs) (21). Concurrently, T-cell and neutrophil infiltration exacerbates neuroinflammation; neutrophils release extracellular traps (NETs), whose DNA payloads activate Absent in Melanoma 2 (AIM2) inflammasomes in endothelial cells, linking innate immunity to vascular damage.

Recent studies have further expanded the inflammatory cytokine network involved in DR and suggest that these mediators act in a retinal cell-type-specific manner. Activated microglia and macrophages are major sources of TNF-α, IL-1β, IL-6, C-C motif chemokine ligand 2(CCL2)/monocyte chemoattractant protein-1 (MCP-1), and reactive nitrogen/oxygen species, thereby amplifying neuroinflammation and promoting secondary activation of Müller cells and endothelial cells. Müller cells are both targets and sources of inflammatory mediators; under hyperglycemic stress, they can produce IL-6, CCL2/MCP-1, VEGF, and other cytokines, contributing to gliosis, neuronal dysfunction, leukocyte recruitment, and BRB disruption (22). Retinal vascular endothelial cells respond to TNF-α, IL-1β, interleukin-17A (IL-17A), CCL2, and C-X-C motif chemokine ligand 1 (CXCL1)/C-X-C motif chemokine ligand 8 (CXCL8) by upregulating adhesion molecules such as intercellular adhesion molecule 1 (ICAM-1) and vascular cell adhesion molecule 1(VCAM-1), which promotes leukostasis, neutrophil recruitment, vascular leakage, and capillary degeneration (23, 24). IL-1β and interleukin-18 (IL-18) reflect NLRP3 inflammasome activation and pyroptosis-related inflammatory amplification in diabetic retinal injury (25). IL-17A has been implicated in non-proliferative DR by enhancing retinal inflammation, oxidative stress, vascular permeability, and capillary degeneration (23). CXCL1-related chemokine signaling contributes to neutrophil recruitment and BRB alteration in DR, linking chemokine activation with innate immune-cell infiltration (24). In advanced proliferative diabetic retinopathy (PDR), single-cell transcriptomic studies of fibrovascular membranes indicate that microglia/macrophages, endothelial cells, stromal or pericyte-like cells, and immune cells participate in inflammatory and fibrotic remodeling (26, 27). Therefore, inflammatory mediators in DR should be interpreted as a dynamic and cell-type-specific network rather than as isolated soluble markers.

### 2.3 Vascular dysfunction and angiogenesis

Hyperglycemia induces endothelial dysfunction, which manifests as enhanced leukocyte adhesion, breakdown of the BRB, and abnormal neovascularization (28, 29). VEGF, upregulated through the Protein Kinase C (PKC)/Mitogen-Activated Protein Kinase (MAPK) signaling pathway, promotes endothelial cell proliferation and migration but also leads to the formation of fragile, leaky vessels (30, 31). The imbalance between pro-angiogenic factors, such as VEGF, and anti-angiogenic factors, including pigment epithelium-derived factor (PEDF), drives proliferative DR (32, 33). In this condition, neovascular tufts that are prone to hemorrhage and fibrosis emerge. Matrix metalloproteinases (MMP2/MMP9), activated by inflammatory signals, degrade basement membranes, further compromising vascular integrity.

### 2.4 Retinal neurodegeneration

Neurodegeneration precedes overt vascular changes in DR, with RGC loss and Müller cell dysfunction serving as early indicators (34). Hyperglycemia impairs axonal transport and mitochondrial dynamics in RGCs, triggering caspase-dependent apoptosis and necroptotic pathways (35, 36). Glutamate excitotoxicity, resulting from impaired glutamate uptake by Müller cells, further damages neurons by overstimulating N-Methyl-D-aspartic Acid (NMDA) receptors (37, 38). Moreover, the classically activated pro-inflammatory macrophage/microglial phenotype (M1) polarization of microglia releases neurotoxic factors, such as nitric oxide (NO), which contribute to RGC degeneration (39). This “neurovascular uncoupling” disrupts retinal function, even in the absence of clinically detectable vasculopathy (40).

Diabetes also disrupts the balance of neurotrophic factors that maintain retinal neuronal and glial survival. Brain-derived neurotrophic factor (BDNF), mainly supported by Müller glia and other retinal cells, is reduced under diabetic or oxidative stress conditions, and reduced BDNF availability may weaken tropomyosin receptor kinase B (TrkB)–protein kinase B (AKT)/extracellular signal-regulated kinase (ERK) survival signaling, thereby increasing the susceptibility of Müller cells and retinal neurons to degeneration (41, 42). Nerve growth factor (NGF) signaling through tropomyosin receptor kinase A (TrkA) also appears to participate in both neuronal and vascular homeostasis, and topical NGF treatment has been reported to improve neurodegenerative and vascular abnormalities in experimental DR, although its clinical translational value still requires further validation (43, 44). Ciliary neurotrophic factor (CNTF) may represent a compensatory neuroprotective or glial stress-related response in early diabetic retinal injury, suggesting a role for Müller cell–RGC trophic support during early neurodegeneration (45). Glial cell line-derived neurotrophic factor (GDNF) has been linked to retinal neuronal protection by improving glutamate handling and reducing neural apoptosis in streptozotocin (STZ)-induced diabetic retina, but DR-specific evidence remains less extensive than that for BDNF or vascular endothelial growth factor receptor 2 (VEGF) (46, 47). PEDF has both anti-angiogenic and neurotrophic properties; diabetes-associated reduction or functional imbalance of PEDF may contribute to retinal inflammation, neuronal cell death, vascular leakage, and VEGF-dominant neovascular signaling (48, 49). Conversely, VEGF has a dual role in DR: excessive VEGF promotes BRB breakdown and pathological neovascularization, whereas Müller cell VEGF/VEGFR2 signaling can support Müller cell viability and BDNF production, indicating that complete or prolonged VEGF suppression may affect neurotrophic homeostasis (41). Therefore, diabetes-induced neurotrophic imbalance may interact with oxidative stress, glutamate excitotoxicity, inflammation, and PANoptosis-related inflammatory cell death to accelerate retinal neurodegeneration.

### 2.5 Altered retinal metabolism

Metabolic derangements are fundamental to the progression of DR, with glucose overload reprogramming retinal energy metabolism towards glycolysis and lipid peroxidation. Advanced glycation end products (AGEs), which are formed through non-enzymatic glycation, activate Receptor for Advanced Glycation Endproducts (RAGE) signaling, thereby promoting inflammation mediated by Nuclear Factor kappa-light-chain-enhancer of activated B cells (NF-κB) and oxidative stress (50). Dysregulated lipid metabolism, characterized by elevated levels of free fatty acids(FFAs), engages the Toll-like Receptor 4 (TLR4)/Myeloid Differentiation primary response gene 88 (MyD88) pathways, driving the activation of the NLRP3 inflammasome in endothelial cells (51, 52).

In addition to altered lipid metabolism, lipoxidation has an important role in diabetic retinal injury. Hyperglycemia-induced ROS promotes lipid peroxidation of polyunsaturated fatty acids in the retina, generating reactive lipid aldehydes such as acrolein, 4-hydroxynonenal (4-HNE), malondialdehyde (MDA), and glyoxal, which can form advanced lipoxidation end products (ALEs) with proteins, phospholipids, and DNA (53). Acrolein and acrolein-derived ALEs, including Nϵ-(3-formyl-3, 4-dehydropiperidino)lysine (FDP-Lysine), have been implicated in diabetic retinal oxidative injury and may induce Müller glial dysfunction, potassium-channel dysregulation, VEGF upregulation, pro-inflammatory cytokine release, and neuronal or vascular cell injury (54). 4-HNE and MDA are also widely used indicators of lipid peroxidation in DR and may contribute to mitochondrial dysfunction, endoplasmic reticulum stress, pericyte injury, endothelial dysfunction, and BRB disruption (53). Lipid peroxidation is also mechanistically linked to ferroptosis, a regulated cell death process characterized by iron-dependent lipid peroxide accumulation and impaired antioxidant defense; recent evidence suggests that ferroptosis-related mitochondrial damage may participate in diabetic retinal injury (55). Therefore, lipoxidation may amplify oxidative stress, inflammatory signaling, and PANoptosis-related inflammatory cell death by promoting mitochondrial damage, NLRP3 inflammasome activation, and lipid peroxide accumulation. Amino acid imbalances, such as decreased taurine availability, impair osmoregulation and antioxidant defenses, further sensitizing retinal cells to stress (56).

### 2.6 Epigenetic modifications

Epigenetic mechanisms intricately regulate the pathogenesis of DR via DNA methylation, histone modification, and ncRNA regulation (57). Hyperglycemia triggers hypomethylation of pro-inflammatory genes (e.g., IL-6) and hypermethylation of antioxidant genes, thereby altering the transcriptional landscape. ncRNAs play pivotal roles as modulators: MicroRNA-34a (miR-34a) enhances Protein 53 (p53) activity by targeting Sirtuin 1 (SIRT1) (58), thereby promoting RGC apoptosis, while Long non-coding RNA Homologous to E6-AP Carboxyl Terminus domain E3 ubiquitin protein ligase 1-antisense RNA 1 (lncRNA HECTD1-AS1) acts as a sponge for MicroRNA-124-3p (miR-124-3p), leading to the upregulation of NLRP3 in endothelial cells (59, 60).

### 2.7 Breakdown of the blood-retinal barrier

The BRB, which consists of endothelial tight junctions and retinal pigment epithelium (RPE) integrity, is disrupted by ROS and cytokines induced by hyperglycemia. TNF-α and VEGF induce endothelial fenestration by downregulating claudin-5 and occludin, permitting albumin and immune cells to extravasate into the retina (61, 62). Pericyte loss is an early hallmark of DR and contributes to microvascular destabilization, acellular capillary formation, endothelial dysfunction, and BRB leakage (63). Hyperglycemia-induced oxidative stress, inflammatory cytokines, mitochondrial dysfunction, and apoptosis- or necroptosis-related pathways may participate in pericyte and endothelial injury (64). A human iBRB-on-a-chip model recently reproduced early DR-like phenotypes, including pericyte loss, vascular regression, ghost vessels, and inflammatory factor production under diabetic stimulation (65). However, whether pericyte dropout in DR is directly mediated by bona fide PANoptosis remains insufficiently demonstrated; therefore, this process should be described as potentially associated with PANoptosis-related inflammatory injury rather than as a confirmed PANoptosis-dependent event.

## 3 Molecular mechanisms of PANoptosis in DR

### 3.1 Interaction regulation of core signaling pathways

The molecular mechanisms of PANoptosis in DR involve intricate crosstalk among multiple signaling pathways, which may contribute to inflammatory cell death and retinal neurodegeneration. (**Table 1**) Central to this process is the MAPK–ROS–Drp1 axis, in which hyperglycemia-induced ERK1/2 phosphorylation promotes Drp1-mediated mitochondrial fission, leading to mitochondrial dysfunction, ROS accumulation, and oxidative damage of mtDNA, which may further facilitate Z-form DNA binding protein 1 (ZBP1)-related inflammatory signaling (79–81). This association is supported by studies showing that inhibition of Drp1 with Mitochondrial Division Inhibitor 1 (Mdivi-1) reduces the expression of cleaved caspase-3, NLRP3, and phosphorylated Mixed Lineage Kinase Domain-Like Protein (p-MLKL) in RGCs, thereby attenuating the co-activation of apoptosis-, pyroptosis-, and necroptosis-related markers (82, 83). Therefore, the ERK1/2–Drp1 axis may exacerbate mitochondrial dysfunction and ROS production, creating a pro-inflammatory milieu that contributes to PANoptosis-related inflammatory cell death signaling in DR, although direct biochemical evidence for complete ZBP1-PANoptosome assembly in diabetic retinal tissues remains to be further established (84).

**Table 1:** Comparison of cell death modes and evidence sources in DR.

Cell death mode	Core mechanism	Key molecules/pathways	Role and manifestation in DR	Association with PANoptosis	Diabetic model/species/evidence source	References
Apoptosis	Caspase-dependent programmed cell death characterized by membrane blebbing and nuclear fragmentation.	Cleaved caspase-3/-8; Bax/Bcl-2; p53	Caspase activation and retinal vascular cell apoptosis have been observed in diabetic retina and may contribute to early capillary degeneration and neurodegeneration.	Apoptosis is one of the three core cell-death modules that can be integrated into PANoptosis; pathway co-activation should be distinguished from complete PANoptosome assembly.	STZ-induced diabetic mice; galactose-fed mice; diabetic human retinal samples.	(66)
Autophagy/mitophagy	Lysosomal degradation of damaged organelles; dysregulated mitophagy may aggravate mitochondrial stress.	LC3; p62; Beclin-1; PINK1/Parkin; TXNIP	Hyperglycemia can disrupt mitochondrial quality control and may promote oxidative stress, mitochondrial dysfunction, and retinal cell apoptosis.	Autophagy/mitophagy dysfunction may provide upstream mitochondrial danger signals that facilitate PANoptosis-related inflammatory signaling, but it does not by itself prove PANoptosome assembly.	High-glucose-treated retinal Müller cells or RPE cells; experimental diabetic retinal injury models.	(67, 68)
Pyroptosis	Inflammasome-mediated inflammatory cell death involving caspase-1 activation, GSDMD pore formation, and IL-1β/IL-18 release.	NLRP3; caspase-1; GSDMD; IL-1β; IL-18; P2X7	High glucose can induce pericyte loss and endothelial inflammatory injury partly through NLRP3-caspase-1-GSDMD signaling.	Pyroptosis is a core component of PANoptosis and may be co-activated with apoptosis and necroptosis; integrated PANoptosis requires evidence of pathway convergence.	High-glucose-treated human retinal pericytes; high-glucose or inflammatory retinal endothelial cell models.	(69, 70)
Necroptosis	RIPK1/RIPK3/MLKL-mediated regulated necrosis with membrane rupture and inflammation.	RIPK1; RIPK3; p-MLKL	High glucose increases RIPK1/RIPK3 expression in RGCs, while RIP3-mediated microglial necroptosis has been implicated in early neuroinflammation and neurodegeneration in DR.	Necroptosis may cooperate with caspase-8 and inflammasome signaling during PANoptosis-like inflammatory cell death; direct complex assembly remains to be confirmed.	High-glucose-cultured RGCs; diabetic mouse retina; high-glucose BV2 microglia models.	(71, 72)
PANoptosis/PANoptosis-like marker co-activation	Coordinated inflammatory cell death involving apoptosis, pyroptosis, and necroptosis, usually associated with PANoptosome-related complexes.	ZBP1; RIPK3; caspase-1/-3/-8; NLRP3; AIM2; GSDMD	DKK1 reduced cleaved GSDMD, caspase-3, RIPK3, VEGF, MMP2/MMP9, and retinal neovascularization in STZ-induced diabetic rats.	Available DR evidence supports PANoptosis-related marker co-activation; complete ZBP1/AIM2-containing PANoptosome assembly in diabetic retina remains insufficiently validated.	STZ-induced diabetic rat retina; retinal ischemia/reperfusion or acute glaucoma models as retinal extrapolation.	(12, 73)
Ferroptosis	Iron-dependent lipid peroxidation-associated regulated cell death caused by impaired antioxidant defense and GPX4 dysfunction.	GPX4; ACSL4; lipid ROS; Nrf2; Fer-1	Ferroptosis contributes to retinal microvascular dysfunction, and ferroptosis inhibition has been reported to reduce retinal microvasculopathy in diabetic mice.	Ferroptosis may share upstream oxidative stress, mitochondrial damage, and lipid peroxidation signals with PANoptosis-related inflammatory injury; proven integration remains unclear.	High-glucose-treated HRMECs; diabetic mouse retina; human PDR fibrovascular membrane samples.	(55, 74)
NETosis	Neutrophil extracellular trap formation with release of extracellular DNA, histones, neutrophil elastase, MPO, and citrullinated histone H3.	NETs; extracellular DNA; NE; MPO; Cit-H3; AIM2/ZBP1	Hyperglycemia promotes NETosis; NET components are increased in serum or ocular tissues from diabetic or PDR patients and may contribute to vascular inflammation.	NET-derived DNA may provide a DNA-sensing stimulus for AIM2/ZBP1-related inflammasome or PANoptosis-like pathways, but direct DR PANoptosome evidence remains limited.	Serum and ocular samples from diabetic/PDR patients; high-glucose neutrophil models; diabetic mouse and human neutrophil studies.	(75–77)
Anoikis	Apoptosis induced by cell detachment from the extracellular matrix and disruption of integrin-mediated survival signaling.	Integrin β1; FAK; PI3K/Akt	Diabetes-induced basement membrane thickening and abnormal ECM-cell adhesion may promote endothelial or pericyte instability, vascular leakage, and capillary degeneration.	Anoikis is not a PANoptosis-specific mechanism, but it may act together with inflammatory cell death by promoting vascular cell loss and BRB disruption.	DR basement membrane thickening studies; high-glucose retinal endothelial cell/ECM interaction evidence.	(78)

DR, diabetic retinopathy; RGCs, retinal ganglion cells; RPE, retinal pigment epithelium; STZ, streptozotocin; HRMECs, human retinal microvascular endothelial cells; PDR, proliferative diabetic retinopathy; BRB, blood-retinal barrier; ECM, extracellular matrix; GSDMD, gasdermin D; MLKL, mixed lineage kinase domain-like protein; MPO, myeloperoxidase; NE, neutrophil elastase; NETs, neutrophil extracellular traps; Cit-H3, citrullinated histone H3; Fer-1, ferrostatin-1.

TNF-α and Interferon-γ (IFN-γ) are inflammatory mediators that may link immune activation to PANoptosis-related inflammatory cell death through Janus Kinase (JAK)/Signal Transducer and Activator of Transcription 1 (STAT1)–Interferon Regulatory Factor 1 (IRF1) and NF-κB signaling (9, 85). In several non-retinal inflammatory disease models, combined TNF-α/IFN-γ stimulation has been shown to induce inducible nitric oxide synthase (iNOS)-dependent nitric oxide production and caspase-8/Fas cell surface death receptor (Fas)-Associated Death Domain protein (FADD)-associated inflammatory cell death (8, 86, 87). (PMID: 37823450). In diabetic retinal tissues and high-glucose retinal cell models, TNF-α-, IFN-γ-, and NF-κB-related inflammatory activation has been reported; however, direct evidence demonstrating that TNF-α/IFN-γ drives complete PANoptosome-dependent cell death in diabetic retinal endothelial cells remains limited (22–24). Therefore, this pathway should be described as an immune-inflammatory axis that may facilitate PANoptosis-related signaling rather than as a fully established DR-specific PANoptosis mechanism.

**Figure 2:**
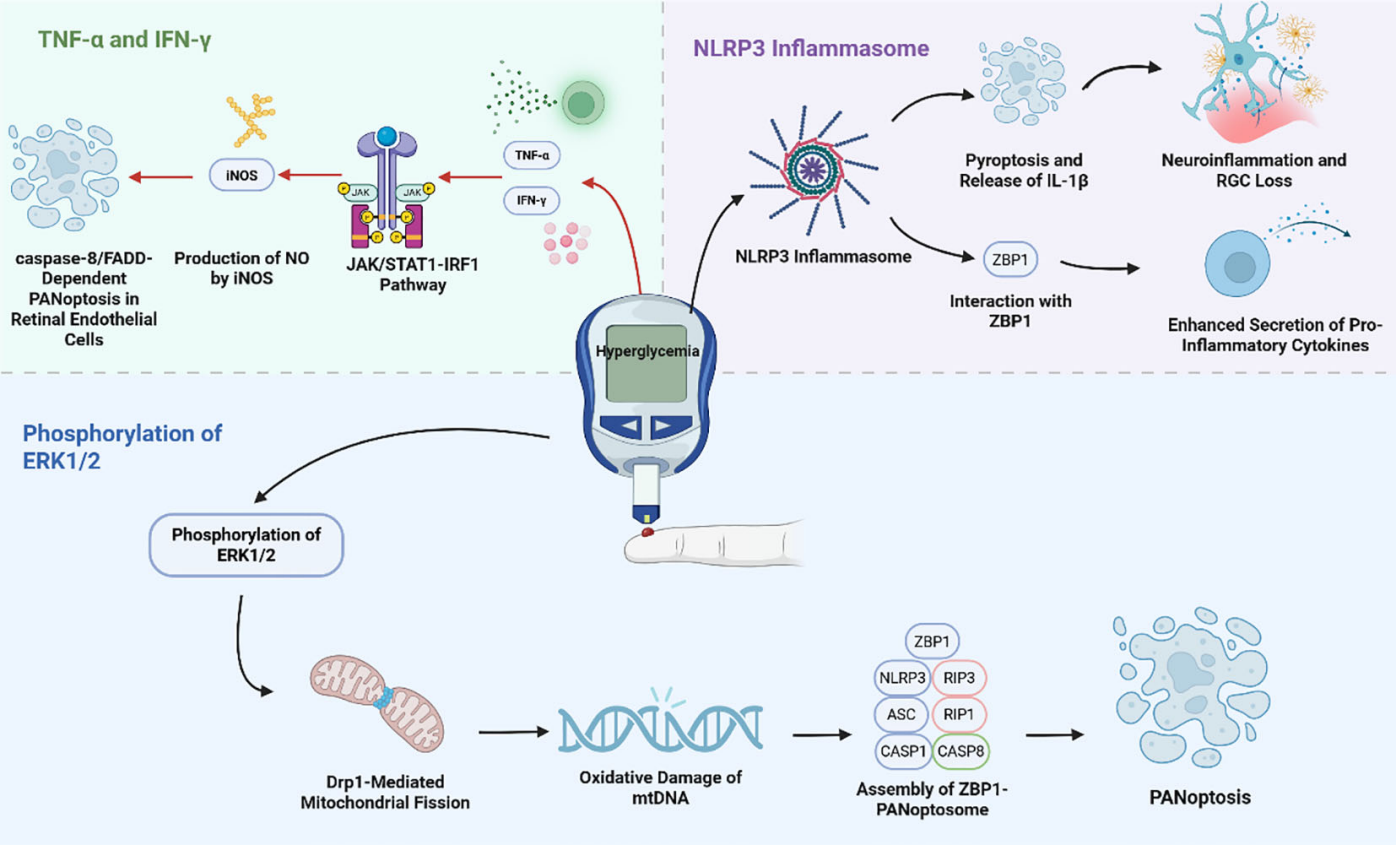
Proposed PANoptosis-related signaling pathways in DR. This figure summarizes candidate signaling pathways that may contribute to PANoptosis-like inflammatory cell death in DR. Hyperglycemia may activate ERK1/2 and promote Drp1-mediated mitochondrial fission, leading to mitochondrial stress and oxidative mtDNA damage. These events may provide upstream danger signals for ZBP1-related inflammatory signaling. High glucose and inflammatory cytokines may also activate TNF-α/IFN-γ–JAK/STAT1–IRF1 signaling and NLRP3 inflammasome pathways, resulting in the co-activation of apoptosis-, pyroptosis-, and necroptosis-related markers. However, direct biochemical evidence confirming complete PANoptosome assembly in diabetic retinal tissues remains limited; therefore, these pathways should be interpreted as proposed PANoptosis-related mechanisms rather than fully established DR-specific PANoptosome pathways.

The NLRP3 inflammasome contributes to pyroptosis-related inflammatory amplification in DR and may interact with ZBP1-associated signaling under inflammatory or DNA-sensing conditions; however, direct evidence for NLRP3–ZBP1-containing PANoptosome assembly in diabetic retinal tissues remains limited (25, 88, 89). Retinal ischemia/reperfusion injury models demonstrate the upregulation of NLRP3, caspase-1, and ZBP1, correlating with exacerbated neuroinflammation and RGC loss (73, 90, 91) (**Figure 2**).

Moreover, activation of the RIPK1/RIPK3–MLKL pathway may contribute to necroptosis-related injury in retinal microglia, RGCs, or vascular cells under diabetic or ischemic stress. Studies in diabetic retinal models have reported increased RIPK3/MLKL-related signaling and microglial neuroinflammatory injury, while retinal ischemia/reperfusion models support a role for RIPK3/MLKL-mediated necroptosis in retinal ganglion cell degeneration (12, 72, 73, 92). However, the extent to which this pathway is integrated into bona fide PANoptosis in DR remains to be determined. In summary, these pathways should be interpreted as interconnected inflammatory cell death signals that may contribute to PANoptosis-related retinal injury, rather than as fully validated DR-specific PANoptosome pathways.

### 3.2 Regulatory networks of non-coding RNAs

The regulatory landscape of ncRNAs in PANoptosis during DR involves intricate interactions between microRNAs (miRNAs), long non-coding RNAs (lncRNAs), and circular RNAs (circRNAs), which modulate cell death pathways and neuroinflammation. MiRNAs play dual roles in the regulation of PANoptosis (93, 94). For example, miR-34a contributes to the crosstalk between apoptosis and pyroptosis by targeting SIRT1, a key deacetylase that typically suppresses p53 activity (58, 59). By inhibiting SIRT1, miR-34a promotes p53 acetylation and activation, resulting in the upregulation of pro-apoptotic genes and pyroptotic markers in RGCs (95). On the other hand, microRNA-181a (miR-181a) exerts protective effects by suppressing the TLR4/MyD88 signaling pathway, reducing the production of pro-inflammatory cytokines and alleviating PANoptosis-associated neuroinflammation (96).

Importantly, these ncRNA interactions should not be interpreted as occurring uniformly in the same retinal cell type. In retinal vascular endothelial cells, the miR-34a/SIRT1 axis has been reported to promote endothelial apoptosis in DR rats, while miR-124-3p regulates high-glucose-induced dysfunction of human retinal microvascular endothelial cells, suggesting that endothelial ncRNA axes are mainly related to apoptosis, proliferation, migration, angiogenic dysfunction, and BRB-related vascular injury (97, 98). In Müller cells, lncRNA KCNQ1 overlapping transcript 1 (KCNQ1OT1) acts as a microRNA-17-5p (miR-17-5p) sponge and regulates thioredoxin-interacting protein (TXNIP)/NLRP3 inflammasome activation under high-glucose conditions, indicating that Müller-cell-related ncRNA pathways may be more closely associated with inflammasome activation and glial inflammatory stress (88). Aquaporin 4 antisense RNA 1(AQP4-AS1)-related mechanisms further suggest that lncRNAs can participate in diabetes-induced retinal neurovascular dysfunction, possibly involving glial–vascular crosstalk rather than a single isolated retinal cell population (99). Therefore, ncRNA–PANoptosis interactions in DR should be interpreted as cell-type- and model-dependent regulatory axes rather than as a uniform mechanism across RGCs, endothelial cells, Müller cells, microglia, and other retinal cell populations.

LncRNAs function as molecular sponges or scaffolds within PANoptosis pathways. For example, the lncRNA HECTD1-AS1 may alleviate the inhibition of miR-124-3p on NLRP3 through adsorption. By reducing the availability of miR-124-3p, HECTD1-AS1 enhances NLRP3 inflammasome assembly in retinal endothelial cells, thereby exacerbating pyroptotic cell death (100, 101). However, its specific mechanism in DR requires further study. Although direct evidence in DR is still forthcoming, this mechanism is deduced from studies indicating HECTD1-AS1-mediated NLRP3 activation in other inflammatory models (10).

CircRNAs also influence PANoptosis via competitive endogenous RNA mechanisms. Circular RNA cerebellar degeneration-related protein 1 antisense RNA (CircRNA CDR1) as binds to microRNA-7 (miR-7), alleviating miR-7-mediated suppression of AIM2, a DNA sensor crucial for PANoptosome formation (102). In DR, elevated CDR1as expression hastens double-stranded DNA (dsDNA)-triggered AIM2 activation, which promotes caspase-1-dependent pyroptosis and caspase-8-dependent apoptosis in RGCs (10, 91).

Emerging evidence suggests that ncRNAs may participate in the regulation of inflammation-associated PCD in retinal and neurovascular injury, but their direct involvement in DR-related PANoptosis remains incompletely defined. For example, microRNA-423-5p (miR-423-5p) has been reported to modulate microglial polarization and neuronal PANoptosis in a cerebral ischemia/reperfusion model by targeting nucleotide-binding oligomerization domain-containing protein 2 (NOD2) and suppressing downstream NF-κB/MAPK signaling; however, this evidence is derived from a non-diabetic cerebral ischemia/reperfusion system and should therefore be interpreted as mechanistic extrapolation rather than direct DR evidence (103). Similarly, retinal ischemia/reperfusion studies have demonstrated PANoptosis-like cell death in retinal neurons, with upregulation of caspase-1, caspase-8, and NLRP3, but these findings do not establish that the same ncRNA-regulated PANoptosome axis operates in diabetic retinal tissues (73). Proposed circRNA–miRNA–RIPK3 or circRNA–miRNA–AIM2 interactions may provide useful hypotheses for linking ncRNA networks to necroptosis, pyroptosis, and apoptosis; nevertheless, most of these axes still require validation in DR-specific models, including high-glucose retinal cells, diabetic animal models, and clinical retinal or vitreous samples.

Collectively, available evidence suggests that ncRNAs may participate in the regulation of PANoptosis-related inflammatory cell death in DR, but the temporal dynamics of these regulatory networks remain insufficiently defined. In early non-proliferative DR, ncRNA-mediated regulation may be more closely associated with microglial activation, Müller cell stress, retinal ganglion cell injury, pericyte loss, and endothelial dysfunction. In contrast, in diabetic macular edema and proliferative DR, ncRNA-related pathways may be more closely linked to BRB disruption, endothelial proliferation, fibrovascular membrane formation, pathological neovascularization, and persistent inflammatory remodeling. Therefore, ncRNA-mediated regulation of PANoptosis-related pathways should be interpreted as cell-type-, model-, and disease-stage-dependent rather than as a uniform mechanism throughout DR progression. Longitudinal single-cell RNA sequencing, spatial transcriptomics, and stage-specific validation using clinical retinal, vitreous, blood, and fibrovascular membrane samples are needed to clarify whether ncRNA–PANoptosis regulatory axes differ between early and late stages of DR (4, 99, 104, 105).

### 3.3 Immune cells and inflammatory microenvironment

**Figure 3:**
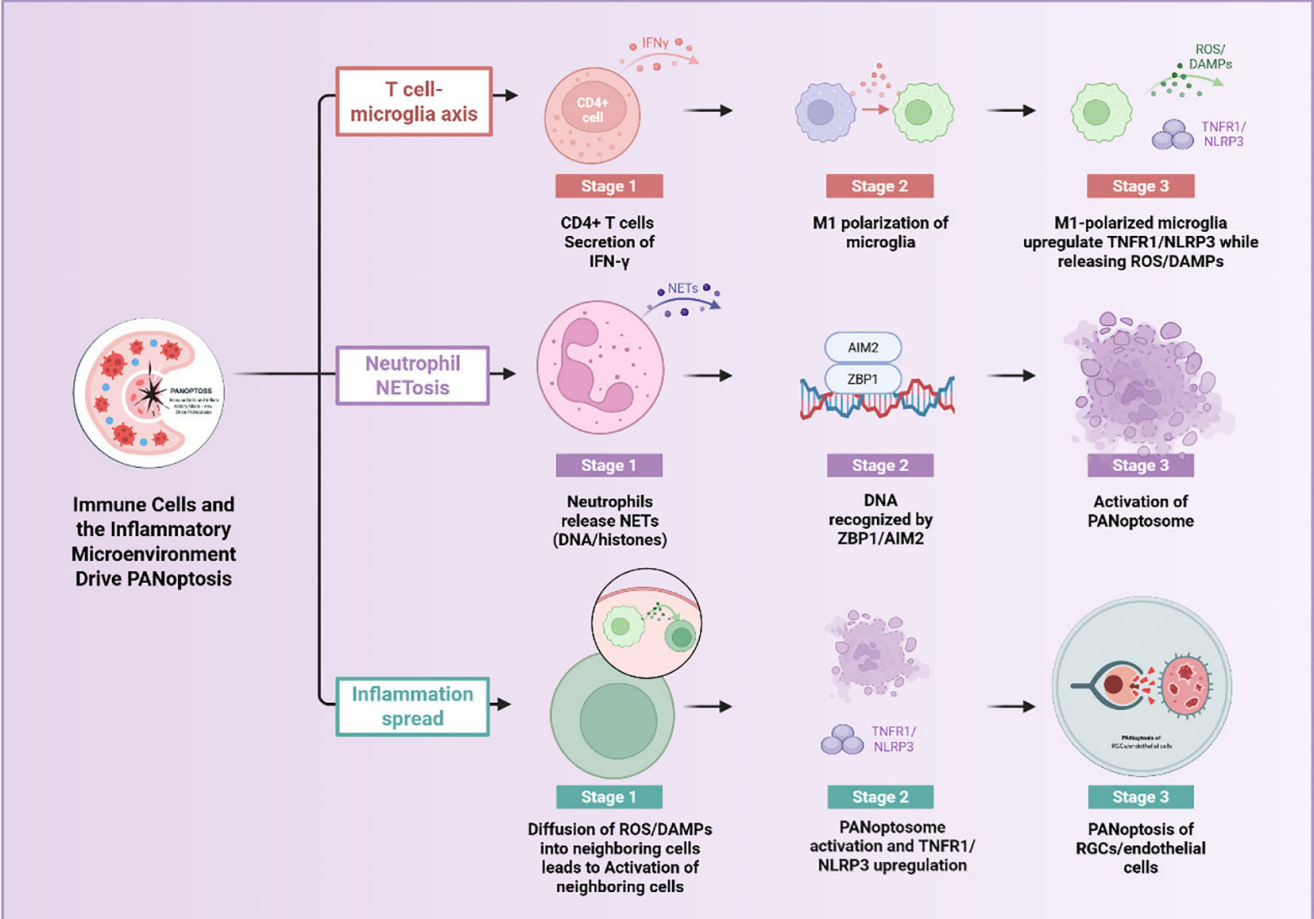
Schematic diagram of immune cells and inflammatory microenvironment driving PANoptosis-related signaling. This figure illustrates a proposed mechanism by which immune cells and the inflammatory microenvironment may contribute to PANoptosis-related inflammatory cell death in DR. In the T cell–microglia axis, CD4^+^ T cells secrete IFN-γ and may promote M1-like microglial polarization. Activated microglia can upregulate TNFR1/NLRP3-related inflammatory signaling and release ROS/DAMPs, forming a positive feedback loop with neighboring retinal cells. Neutrophils release NETs containing DNA and histones via NETosis, which may provide extracellular DNA-related danger signals for AIM2- or ZBP1-associated inflammatory pathways; however, direct evidence that NET-derived DNA induces complete PANoptosome assembly in diabetic retinal cells remains limited. During inflammatory spread, ROS, DAMPs, cytokines, and extracellular DNA signals may diffuse into neighboring cells and promote apoptosis-, pyroptosis-, and necroptosis-related marker co-activation. Therefore, this figure should be interpreted as a proposed immune–inflammation–PANoptosis-related signaling model rather than a fully established DR-specific PANoptosome pathway.

The interplay between immune cells and the inflammatory microenvironment may contribute to PANoptosis-related inflammatory signaling in DR (4). Activated Cluster of Differentiation 4-positive T lymphocytes (CD4+ T cells) secrete IFN-γ, which promotes microglial M1 polarization—a pro-inflammatory state characterized by increased production of TNF-α and IL-6 (106). This establishes a positive feedback loop: IFN-γ-induced microglia may enhance PANoptosis-related inflammatory signaling by upregulating pro-death receptors (e.g., Tumor Necrosis Factor Receptor 1 (TNFR1)) and inflammasome components (e.g., NLRP3) in retinal neurons and endothelial cells (107, 108). At the same time, M1-polarized microglia release ROS and DAMPs, further activating PANoptotic pathways in neighboring cells (109). Evidence supporting the CD4+ T cell/IFN-γ–microglia axis comes mainly from ocular neuroinflammatory models rather than direct DR-specific PANoptosis models. In a mouse retinal ischemia/reperfusion model, CD4+ T-cell infiltration and microglial activation accompanied progressive RGC loss, and T-cell deficiency or IFN-γ neutralization attenuated RGC degeneration and retinal function loss (110). In a diabetic mouse corneal nerve degeneration model, CD4+CD25− T cells secreted IFN-γ, and IFN-γ blockade alleviated trigeminal ganglion neurite inhibition and corneal nerve degeneration (111). Therefore, these findings support the concept that CD4+ T cell/IFN-γ-mediated immune crosstalk may contribute to ocular neuroinflammatory injury under ischemic or diabetic conditions, but they should be interpreted as retinal or ocular extrapolation rather than direct proof of PANoptosis-related retinal damage in DR. (**Figure 3**).

NETosis may provide an additional inflammatory input for PANoptosis-related signaling in diabetic retinal injury. Under diabetic conditions, neutrophil activation and NET release can increase extracellular DNA, histones, neutrophil elastase, myeloperoxidase, and other proteases, which may aggravate endothelial inflammation, leukostasis, vascular leakage, and BRB disruption (75, 112, 113). In infection-related inflammatory models, ZBP1 has been reported to activate inflammasome/PANoptosome signaling, and in sepsis-related myocardial injury models, mtDNA efflux has been linked to AIM2-mediated ZBP1-PANoptosome activation (76, 77, 114). Therefore, NET-derived extracellular DNA may theoretically provide DNA-sensing stimuli for AIM2- or ZBP1-associated inflammatory pathways in DR; however, direct evidence that NET-derived DNA induces complete PANoptosome assembly in diabetic retinal endothelial cells remains limited.

### 3.4 Roles of novel PANoptosome complexes

ZBP1-and AIM2-associated PANoptosome complexes provide a useful mechanistic framework for linking mitochondrial damage, cytosolic DNA sensing, and inflammatory cell death in retinal disease. In the diabetic retina, hyperglycemia-induced mitochondrial dysfunction, oxidative mtDNA damage, impaired mitophagy, NET-derived DNA, and dying-cell-derived DNA may theoretically provide upstream danger signals for ZBP1- or AIM2-related inflammatory signaling. However, the extent to which complete ZBP1- or AIM2-containing PANoptosome assembly occurs in DR-specific models remains incompletely defined.

Current DR-related evidence mainly supports PANoptosis-related marker co-activation rather than definitive biochemical assembly of ZBP1- or AIM2-PANoptosomes. In STZ-induced diabetic rats, Dickkopf-1 (DKK1) administration reduced cleaved gasdermin D (GSDMD), caspase-3, RIPK3, VEGF, MMP2/MMP9, and retinal neovascularization, supporting the presence of PANoptosis-related inflammatory cell death signatures in DR; however, this study did not directly demonstrate ZBP1- or AIM2-containing PANoptosome assembly (12). Retinal ischemia/reperfusion models have shown PANoptosis-like cell death in retinal neurons with activation of pyroptosis-, apoptosis-, and necroptosis-related markers, but these data represent non-diabetic retinal injury rather than direct DR evidence (73). Oxidative-stress studies in retinal pigment epithelial cells further support a retinal DNA-sensing mechanism in which damaged mtDNA can activate ZBP1-related inflammatory responses in microglia, but this evidence is not specific to diabetic retinal PANoptosome formation (115).

By contrast, stronger biochemical evidence for AIM2-, ZBP1-, and related PANoptosome complexes comes mainly from infectious or systemic inflammatory models. AIM2 has been shown to form a multiprotein complex with pyrin, ZBP1, apoptosis-associated speck-like protein containing a CARD (ASC), caspase-1, caspase-8, RIPK1, RIPK3, and FADD during host defense against herpes simplex virus 1 and Francisella novicida infection (11). Additional studies have shown that IRF1 can contribute to ZBP1-, AIM2-, RIPK1-, and NOD-like receptor family pyrin domain-containing 12 (NLRP12)-PANoptosome activation, and that integrated activation of multiple inflammasome sensors can drive PANoptosis under inflammatory conditions (5, 116). Therefore, in the context of DR, ZBP1- and AIM2-associated PANoptosomes should be interpreted as promising but incompletely validated mechanisms. Future DR-specific studies should use co-immunoprecipitation, proximity ligation assay, colocalization analysis combined with loss-of-function experiments, and rescue experiments to distinguish bona fide PANoptosome assembly from parallel activation of apoptosis, pyroptosis, and necroptosis markers.

From an experimental perspective, confirmation of PANoptosome assembly generally requires evidence of physical complex formation together with downstream functional activation. In mechanistic PANoptosome studies, assembly has typically been assessed using immunoprecipitation or co-immunoprecipitation to detect interactions among sensor, adaptor, and effector molecules; immunoblotting to evaluate cleavage or activation of caspase-1, caspase-3, caspase-8, GSDMD, RIPK1, RIPK3, and MLKL; immunofluorescence or colocalization analysis to visualize complex-associated proteins; and genetic loss-of-function approaches to determine whether deletion of key components attenuates inflammatory cell death. For example, AIM2–pyrin–ZBP1 PANoptosome formation was supported by evidence that AIM2, pyrin, ZBP1, ASC, caspase-1, caspase-8, RIPK1, RIPK3, and FADD formed a multiprotein complex during host defense against herpes simplex virus 1 (HSV-1) and Francisella novicida infection (11). IRF1-related PANoptosome activation has also been investigated using live-cell imaging, Western blotting, enzyme-linked immunosorbent assay (ELISA), and inflammasome/PANoptosome activation models (5). Integrated inflammasome activation studies further showed that simultaneous activation of multiple inflammasome sensors can drive PANoptosis under inflammatory conditions (116). In contrast, DR-related or retinal injury studies have generally provided marker-level evidence, including changes in cleaved GSDMD, caspase-1, caspase-3, caspase-8, RIPK3, MLKL, NLRP3, IL-1β, and IL-18, rather than direct biochemical confirmation of ZBP1- or AIM2-containing PANoptosome assembly (12, 73). Therefore, future DR-specific studies should use co-immunoprecipitation, proximity ligation assay, confocal colocalization, component-specific knockdown or knockout, and rescue experiments to distinguish bona fide PANoptosome assembly from parallel activation of apoptosis, pyroptosis, and necroptosis markers.

Accurate interpretation of PANoptosis in retinal disease requires distinguishing integrated PANoptosis from concurrent but independent activation of apoptosis, pyroptosis, and necroptosis pathways. In DR-related studies, decreased cleaved GSDMD, caspase-3, RIPK3, VEGF, MMP2, MMP9, and retinal neovascularization after DKK1 treatment supports PANoptosis-related marker modulation, but does not by itself confirm same-cell PANoptosome assembly (12). Similarly, retinal ischemia/reperfusion studies have reported PANoptosis-like neuronal death with activation of pyroptosis-, apoptosis-, and necroptosis-related markers, but these findings were obtained in non-diabetic retinal injury models and should not be directly equated with DR-specific PANoptosome formation (73). By contrast, studies that define bona fide PANoptosis generally demonstrate multiprotein complex formation and functional dependence on core components, as shown for AIM2–pyrin–ZBP1 PANoptosome formation during infectious host defense (11). IRF1-related PANoptosome studies also used functional readouts such as live-cell imaging, Western blotting, and ELISA to support integrated inflammasome/PANoptosome activation rather than isolated pathway activation (5). Therefore, when DR studies rely mainly on bulk retinal tissue or marker-level measurements, simultaneous changes in cleaved caspase-3, caspase-8, GSDMD, NLRP3, RIPK3, MLKL, IL-1β, and IL-18 should be interpreted cautiously as “PANoptosis-related” or “PANoptosis-like marker co-activation.

### 3.5 Crosstalk between metabolic abnormalities and PANoptosis

Metabolic dysregulation in DR fosters a pro-PANoptotic microenvironment by intricately interacting with mitochondrial function and inflammatory signaling. Hyperglycemia drives a “glucose toxicity-mitochondrial dysfunction-PANoptosis” axis by impairing Function 14 Domain Containing 1 (FUNDC1)-mediated mitophagy, a process essential for the clearance of damaged mitochondria (117, 118). Under high glucose conditions, reduced FUNDC1 expression hinders the removal of oxidatively damaged mitochondria, resulting in increased release of mtDNA into the cytoplasm (119, 120). This mtDNA acts as a danger signal to activate ZBP1-PANoptosomes, facilitating the assembly of RIPK3, caspase-8, and NLRP3 complexes in the retina. Concurrently, hyperglycemia intensifies O-Glucosamine N-Acetyl (O-GlcNAc) modification through the hexosamine pathway, post-translationally modifying RIPK3 to promote its phosphorylation and necroptotic signaling, thus further connecting glucose toxicity to PANoptosis (121, 122).

Lipid metabolic abnormalities exacerbate PANoptosis through FFAs-induced TLR4/MyD88 activation (123, 124). Elevated levels of FFAs in diabetic retinas bind to TLR4, triggering MyD88-dependent recruitment of NLRP3 inflammasomes and subsequent caspase-1 activation, which drives the pyroptotic release of IL-1β and IL-18. Furthermore, FFAs activate the Peroxisome Proliferator-Activated Receptor Gamma (PPARγ)-Retinoid X Receptor Alpha (RXRα) pathway, promoting the formation of ASC specks—key components of PANoptosomes—that link apoptosis and pyroptosis (125). This mechanism was validated in animal models where PPARγ agonists exacerbated retinal endothelial cell death, while TLR4 antagonists attenuated NLRP3-driven inflammation (126, 127). Collectively, metabolic disruptions in glucose and lipid homeostasis converge to amplify PANoptosis by compromising mitochondrial quality control, enhancing inflammasome activation, and fostering a pro-inflammatory retinal microenvironment.

## 4 Relationship between retinal neovascularization and PANoptosis

### 4.1 Retinal vascular endothelial cell death and abnormal neovascularization

In the pathological progression of DR, retinal vascular endothelial cell death and abnormal neovascularization are critical events intertwined with PANoptosis, a novel form of cell death that integrates apoptosis, necroptosis, and pyroptosis. Chronic hyperglycemia in diabetes induces endothelial dysfunction by activating multiple death signaling pathways, including PANoptosis, which accelerates endothelial cell loss and disrupts retinal vascular homeostasis (122, 128). Unlike classical apoptosis, PANoptosis in retinal endothelial cells is characterized by the co-activation of caspase-3 (apoptosis), NLRP3 inflammasome (pyroptosis), and RIPK3/MLKL (necroptosis), all of which are exacerbated by diabetic stressors such as oxidative stress and inflammatory cytokines (12, 92).

VEGF, a key mediator of neovascularization, is closely associated with PANoptosis in DR. Hyperglycemia enhances VEGF expression via NF-κB signaling, which stimulates endothelial cell proliferation and concurrently triggers PANoptotic cell death (129, 130). This paradoxical effect fosters a pro-angiogenic microenvironment characterized by excessive vascular sprouting and compromised endothelial survival, resulting in the formation of fragile, leaky new blood vessels. MMP2 and MMP9, whose expression is linked to PANoptosis activation, further degrade the BRB by cleaving basement membrane components, thereby facilitating neovascularization (12).

Experimental models validate that inhibiting PANoptosis—through caspase-8 inhibitors or RIPK3 knockdown—reduces endothelial cell death and attenuates retinal neovascularization (9, 131). For example, blocking the ZBP1-PANoptosome complex in mice decreases VEGF secretion and MMP activity, restoring vascular integrity (132, 133). These findings underscore PANoptosis as a driver of both endothelial cell loss and aberrant angiogenesis in DR.

### 4.2 Regulation of PANoptosis and neovascularization by DKK1

DKK1, an antagonist of the Wingless-type MMTV Integration Site Family (Wnt)/β-catenin pathway, has been implicated as a potential regulator of PANoptosis-related inflammatory cell death and retinal neovascularization in DR. Reduced DKK1 expression in the vitreous of early-stage DR patients has been associated with enhanced Wnt signaling, which may contribute to inflammatory cell death by modulating RIPK3- and NLRP3-related pathways (134, 135). In STZ-induced diabetic rats, intravitreal administration of DKK1 reduced PANoptosis-related marker activation, as reflected by decreased levels of cleaved GSDMD, caspase-3, and p-RIPK3 in retinal tissues (12).

Mechanistically, DKK1 may attenuate inflammatory cell death and retinal neovascularization partly through inhibition of Wnt/β-catenin-related signaling and downregulation of angiogenesis-associated molecules, including VEGF, MMP2, and MMP9. In the original study by Xu et al., DKK1 inhibited Wnt/β-catenin/low-density lipoprotein receptor-related protein 5/6(LRP5/6) signaling, reduced VEGF, MMP2, and MMP9 expression, and decreased retinal neovascularization and acellular vessels in STZ-induced diabetic rats. *In vitro*, DKK1 also repressed the proliferative and migratory ability of endothelial cells by inhibiting angiogenesis-related molecules. Therefore, these findings suggest that DKK1 may link Wnt/β-catenin inhibition with reduced PANoptosis-related marker co-activation and pathological angiogenesis in DR. However, the available evidence mainly supports attenuation of pyroptosis-, apoptosis-, and necroptosis-related markers, rather than definitive proof that DKK1 directly blocks complete PANoptosome assembly in diabetic retinal tissues (12).

## 5 Biomarkers and diagnostic implications of PANoptosis in DR

The identification of PANoptosis-related biomarkers may provide a useful but still preliminary framework for understanding molecular heterogeneity in DR. A recent integrative bioinformatic study identified a six-gene PANoptosis-related signature, including Brain Expressed X-Linked 2 (BEX2), Caspase-2 (CASP2), Cluster of Differentiation 36 (CD36), Fatty Acid Synthase (FASN), Oncostatin M Receptor (OSMR), and Phospholipid Scramblase 3 (PLSCR3), as a candidate diagnostic model for DR (4). Importantly, the specimen source should be explicitly defined. The discovery dataset GSE221521 was based on peripheral blood RNA-seq profiles from healthy controls, diabetic patients without DR, and patients with DR, whereas additional validation involved the GSE60436 dataset, which contains fibrovascular membrane samples from patients with PDR, as well as experimental validation using clinical samples and high-glucose-treated endothelial cells. Therefore, this six-gene signature should be interpreted primarily as a candidate blood transcriptomic and tissue-associated biomarker model. Its potential clinical utility for DR diagnosis, prognosis, disease-stage monitoring, or treatment-response prediction still requires further validation in independent multicenter cohorts, longitudinal studies, clearly defined specimen types, and, where applicable, protein-level assays.

Inflammatory mediators of PANoptosis further enhance diagnostic precision. Elevated serum concentrations of cleaved caspase-1 and IL-1β—key effectors of pyroptosis—correlate with retinal ganglion cell loss and neuroinflammation in DR (136, 137). Although direct validation in DR is ongoing, studies in inflammatory ocular disorders indicate that NETs, DNA-histone complexes released during PANoptosis, are detectable in the vitreous humor and correlate with BRB disruption (90, 138).

Single-cell RNA sequencing (scRNA-seq) has revealed cell-type-specific PANoptosis patterns in DR retinas. Activated microglia and retinal endothelial cells exhibit enriched expression of PANoptosis-associated genes, while Müller glial cells display the highest transcript levels of ZBP1 and RIPK3, spatially coinciding with glial scarring and neurovascular unit damage. Transcriptomics maps PANoptosis hotspots to perivascular and ganglion cell layers, overlapping with regions of vascular leakage and neurodegeneration—patterns directly observed in Amyloid-β peptide 1-40 (Aβ1-40)-induced age-related macular degeneration (AMD) models, where AIM2-PANoptosome activation drives endothelial and retinal pigment epithelial cell death (139). While direct DR-specific data remain limited, these cross-disease model insights highlight PANoptosis as a potential driver of neurovascular dysfunction, with high-resolution technologies linking molecular signatures to structural pathology in retinal degenerative conditions.

Functionally, BEX2 modulates inflammation and cell death pathways, while CASP2 drives apoptotic signaling in hyperglycemic retinal neurons (66, 140, 141). CD36 and FASN highlight lipid metabolic dysregulation in DR, with CD36 mediating free fatty acid uptake and FASN promoting pro-inflammatory lipid synthesis (142). OSMR and PLSCR3 regulate cell membrane integrity and phosphatidylserine exposure, respectively, both of which are critical for PANoptosome assembly (143).

Collectively, these candidate biomarkers may provide a preliminary framework for linking PANoptosis-related molecular signatures with DR phenotypes. Machine learning-derived gene signatures may assist in early detection or risk stratification after external validation. Meanwhile, inflammatory mediators and single-cell/spatial technologies may help define the cellular and spatial contexts of retinal inflammatory cell death.The six-gene signature should therefore be interpreted as a candidate biomarker model with transcriptomic and experimental support. However, further multicenter, longitudinal, and specimen-specific validation is required before these biomarkers can be used for DR prognosis, treatment-response prediction, or clinical decision-making.

## 6 Therapeutic strategies and translational research targeting PANoptosis

Because most PANoptosis-targeted strategies in DR remain at the preclinical or hypothesis-generating stage, therapeutic claims in this section are interpreted according to the model in which each intervention was tested. We distinguish direct DR animal evidence, such as STZ-induced diabetic rats or mice and db/db mice, from high-glucose retinal cell models, human transcriptomic or PDR fibrovascular membrane datasets, non-diabetic retinal injury models, and non-ocular inflammatory or tumor models. Accordingly, small-molecule inhibitors, RNA-based approaches, nanomedicine, extracellular vesicle-based delivery, and immunomodulatory interventions are discussed as potential or exploratory strategies rather than established DR therapies. Melatonin-related studies were mainly performed in STZ-induced diabetic mice and high-glucose-treated neuronal cells (144), whereas DKK1 studies were conducted in STZ-induced diabetic rats receiving intravitreal administration (12). In contrast, several PANoptosis-related nanozyme or immunomodulatory studies were derived from tumor, cerebral ischemia/reperfusion, glaucoma-related, or other non-DR systems and should therefore be interpreted cautiously when extrapolated to DR (103, 145, 146).

### 6.1 Small-molecule inhibitors and natural compounds

Pharmacological interventions aimed at PANoptosis-related pathways in DR have primarily focused on inhibiting necroptosis-related kinases, inflammasome-associated inflammatory cell death, and mitochondrial dysfunction, but the strength of evidence differs markedly according to the experimental model used.

Necrostatin-1, a selective RIPK1 kinase inhibitor, has shown protective effects against RGC injury mainly in glutamate-induced excitotoxic glaucoma models, including glutamate-treated RGC-5 cells *in vitro* and intravitreal glutamate-injected mouse eyes *in vivo*, where it inhibited RIPK1/RIPK3/MLKL-mediated necroptosis and reduced NLRP3 inflammasome activation (71, 145, 147, 148). Notably, similar protective effects of Necrostatin-1 on retinal structure and function have been observed in models of ischemia/reperfusion injury, where it attenuates RIPK1-mediated neuronal loss and vascular leakage (149). However, these ischemia/reperfusion and glaucoma-related models are non-diabetic retinal injury models; therefore, they support the retinal neuroprotective potential of RIPK1 inhibition but should not be interpreted as direct evidence for PANoptosis-specific therapy in DR.

Additional evidence comes from an Aβ1-40-induced AMD model, in which Aβ1–40 was administered to mice to induce retinal-choroidal complex injury *in vivo* and ARPE-19 cells were exposed to Aβ1–40 *in vitro*; in that study, Aβ1–40 increased AIM2-, pyrin inflammasome sensor (PYRIN-), ZBP1-, ASC-, NLRP3-, caspase-, GSDMD-, RIPK1/RIPK3-, and MLKL-related markers, while regulated cell death inhibitors including Necrostatin-1 attenuated Aβ1-40-induced RPE cell injury (139, 150). Thus, Necrostatin-1-related strategies may attenuate necroptosis-associated retinal injury and PANoptosis-like marker co-activation in non-diabetic retinal degeneration models, but DR-specific animal studies confirming suppression of complete PANoptosome assembly remain insufficient.

**Figure 4:**
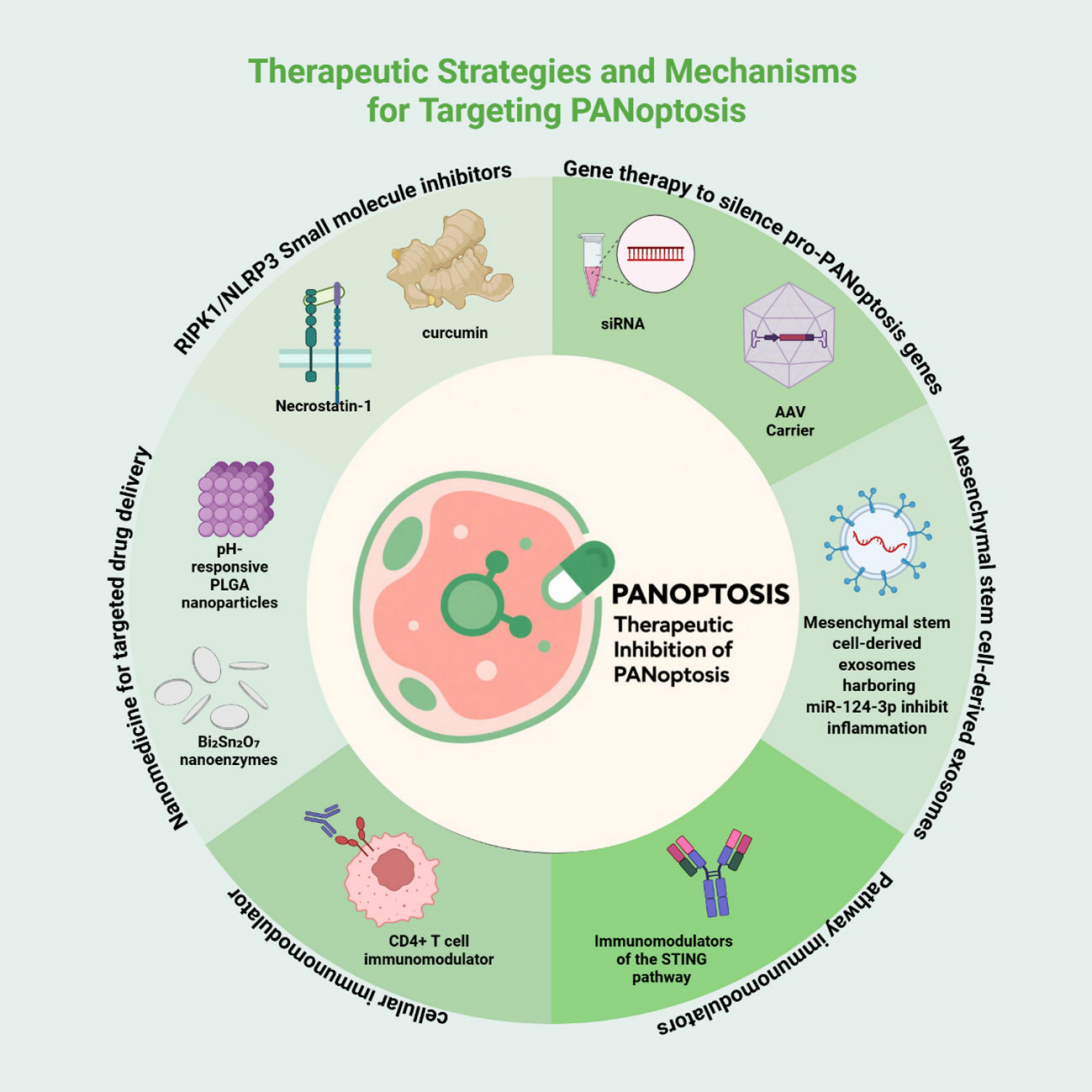
Proposed therapeutic strategies targeting PANoptosis-related pathways in DR. This figure summarizes exploratory therapeutic strategies that may modulate PANoptosis-related inflammatory signaling in DR, including small-molecule or natural compounds, RNA- or gene-based interventions, nanomedicine and extracellular vesicle-based delivery, cell-free mesenchymal stem cell (MSC)-derived exosome approaches, and immunomodulatory strategies. These approaches are interpreted according to their evidence sources, including diabetic animal models, high-glucose retinal cell systems, retinal ischemia/reperfusion or glaucoma-related models, and non-ocular inflammatory or tumor models. Because direct evidence for complete PANoptosome inhibition in diabetic retinal tissues remains limited, these strategies should be viewed as potential or hypothesis-generating interventions rather than established DR therapies.

Similarly, Necrosulfonamide, an inhibitor of MLKL oligomerization, should be discussed primarily as an anti-necroptotic compound rather than as a validated DR therapy. Existing evidence for Necrosulfonamide is mainly derived from non-retinal ischemic models, such as transient middle cerebral artery occlusion (tMCAO) rats *in vivo* and oxygen-glucose deprivation/reoxygenation (OGD/Re)-treated primary rat astrocytes or human astrocytes *in vitro*, where it reduced MLKL/RIPK3-associated necroptotic injury (151–153) (**Figure 4**).

Natural compounds may provide multitargeted regulation of oxidative stress, inflammation, and regulated cell death, but their model specificity should also be clearly defined. Curcumin, a bioactive polyphenol, has been reported to inhibit inflammatory signaling and microglial M1 polarization in neuroinflammatory models, partly through suppression of the TLR4/NF-κB axis and NLRP3 inflammasome activation (154, 155). However, the evidence linking curcumin to PANoptosis is mainly derived from cerebral ischemia/reperfusion models rather than DR-specific retinal models. In cerebral ischemia/reperfusion injury models, curcumin-preconditioned oral MSCs enhanced miR-423-5p-mediated regulation of microglial polarization and reduced NOD2/NF-κB/MAPK-associated neuronal PANoptosis-like injury (103). Therefore, curcumin should be described as a potential anti-inflammatory and neuroprotective compound with mechanistic relevance to PANoptosis-like injury, rather than as a PANoptosis-targeted therapy already validated in diabetic retinal models.

Melatonin, a potent antioxidant, exerts neuroprotective effects by inhibiting mitochondrial reactive oxygen species (mtROS) production and blocking the NLRP3/caspase-1 pathway (156, 157). In STZ-induced diabetic mice and high-glucose-treated neuronal cells, melatonin reduced neuronal death, NLRP3, cleaved caspase-1, N-terminal fragment of gasdermin D (GSDMD-N), IL-1β, microtubule-associated protein 1 light chain 3 (LC3), Beclin-1, and autophagy-related protein 12 (ATG12), suggesting inhibition of neuronal pyroptosis and excessive autophagy under diabetic or high-glucose conditions (138, 144, 158). Thus, melatonin provides relatively direct diabetic-model evidence for neuroprotection against pyroptosis/autophagy-related injury, although direct inhibition of PANoptosome assembly in diabetic retina has not yet been demonstrated.

Vitamin E analogs, by inhibiting Drp1-mediated mitochondrial fission, may disrupt the ERK1/2–Drp1–ROS axis, which has been proposed as an upstream mitochondrial stress pathway associated with PANoptosis-related inflammatory signaling in diabetic retinal injury models (82). However, this mechanism should be interpreted as upstream mitochondrial modulation rather than direct evidence that Vitamin E analogs inhibit PANoptosome assembly in DR.

DKK1, an antagonist of the Wnt/β-catenin pathway, provides more direct DR animal evidence. In STZ-induced diabetic rats, intravitreal DKK1 administration reduced cleaved GSDMD, caspase-3, RIPK3, VEGF, MMP2/MMP9, retinal neovascularization, and acellular vessels, supporting both anti-inflammatory cell-death modulation and anti-neovascular effects in a diabetic retinal model (12). Nevertheless, these findings should be interpreted as PANoptosis-related marker modulation rather than definitive evidence of complete ZBP1- or AIM2-containing PANoptosome suppression in DR.

### 6.2 Gene therapy

Gene therapy has emerged as a potential strategy for long-term modulation of pathogenic pathways in DR, but its application to PANoptosis-specific intervention remains largely exploratory. RNA interference (RNAi) represents one of the most direct approaches for regulating disease-associated gene expression. Small interfering RNA (siRNA) targeting PANoptosis-associated genes, such as FASN and PLSCR3, has shown efficacy in suppressing endothelial cell hyperproliferation and abnormal neovascularization. However, the available evidence for FASN and PLSCR3 should be interpreted according to the experimental source. These genes were mainly identified from peripheral blood transcriptomic datasets and validated using PDR fibrovascular membrane samples, clinical blood samples, high-glucose-treated Human Umbilical Vein Endothelial Cells (HUVECs), and HUVEC knockdown assays rather than diabetic animal gene-therapy models (4, 159). Therefore, siRNA-mediated FASN or PLSCR3 knockdown currently supports candidate target validation in endothelial-cell functional assays, but it should not be described as a gene therapy already validated in diabetic retinal animal models.

Viral vector-based gene therapy provides a broader platform for long-term intraocular gene delivery, but its current relevance to PANoptosis inhibition in DR remains mostly conceptual. In the context of DR research, adeno-associated virus (AAV) vectors, known for their relatively low immunogenicity and ability to achieve long-term gene expression, have emerged as useful tools for retinal gene delivery. Nevertheless, direct DR studies using AAV vectors to deliver dominant-negative ZBP1 or NLRP3 mutants have not been sufficiently established. Therefore, the previous discussion of AAV-mediated dominant-negative ZBP1 or NLRP3 delivery should be reframed as a hypothetical strategy rather than as a validated DR therapeutic approach. More DR-relevant gene-therapy concepts include hyperglycemia-responsive TXNIP promoter-based constructs or TXNIP/NLRP3-related modulation, because TXNIP expression is induced in high-glucose retinal cell culture and diabetic rodent retinas, and *in vivo* inhibition of TXNIP has been reported to block early pathological changes of DR (160–164). However, before clinical translation, such approaches still require validation of vector tropism, duration of transgene expression, retinal cell-type specificity, inflammatory safety, and whether they actually suppress PANoptosis-related pathway integration rather than isolated inflammasome activation.

RNAi-based approaches in DR have expanded to include ncRNAs, with miR-423-5p emerging as a key player. However, the model source of miR-423-5p-related evidence should be clearly distinguished. This miRNA targets NOD2 to inhibit microglial M1 polarization, a process central to retinal inflammation in DR (103, 165). The strongest evidence for miR-423-5p-mediated regulation of PANoptosis-like neuronal injury currently comes from cerebral ischemia/reperfusion models, in which curcumin-preconditioned oral MSCs increased miR-423-5p levels, inhibited NOD2/NF-κB/MAPK signaling, regulated microglial polarization, and reduced neuronal PANoptosis-like injury (103). Therefore, miR-423-5p/NOD2 should be described as a mechanistically relevant ncRNA axis that may inform future DR studies, rather than as an RNAi-based therapy already tested in diabetic retinal animal models. Future DR-focused RNA or gene therapy studies should validate these targets in high-glucose retinal endothelial cells, Müller cells, microglia, RGCs, STZ-induced diabetic rodents, db/db mice, or PDR clinical samples, while also confirming whether the intervention modulates bona fide PANoptosis rather than parallel activation of individual cell-death pathways.

### 6.3 Nanomedicine-based delivery systems

Nanomedicine-based delivery systems may improve local ocular delivery and reduce systemic exposure, but their direct application to PANoptosis inhibition in DR remains insufficiently validated. Among available studies, small extracellular vesicle-loaded bevacizumab provides more direct DR-relevant evidence by reducing VEGF, exudates, leukostasis, and retinal cell death in a rat model of DR; however, this strategy should be interpreted as anti-VEGF nanodelivery rather than PANoptosis-specific therapy (166). In contrast, nanozymes or co-delivery systems designed to manipulate PANoptosis are mainly derived from non-DR or non-ocular models and should be described as hypothesis-generating strategies.

Several original DR-related extracellular vesicle studies further support the value of nanomedicine-based delivery in modulating retinal inflammation, apoptosis, oxidative stress, and angiogenesis. MSC-derived small extracellular vesicles delivering neural precursor cell expressed developmentally downregulated 4 (NEDD4) have been reported to alleviate DR-related retinal injury in STZ-induced diabetic rats and high-glucose-treated ARPE-19 cells by regulating the phosphatase and tensin homolog (PTEN)/AKT/nuclear factor erythroid 2-related factor 2 (NRF2) pathway and reducing apoptosis and oxidative stress (167). Engineered MSC-derived small extracellular vesicles also improved retinal function and alleviated retinal apoptosis, inflammation, and angiogenesis in db/db mice and STZ-induced diabetic rats through hypoxia-inducible factor 1 alpha (HIF-1α)/enhancer of zeste homolog 2 (EZH2)/peroxisome proliferator-activated receptor gamma coactivator 1-alpha (PGC-1α)-related mechanisms (168). In addition, MSC-derived small extracellular vesicles delivering microRNA-22-3p (miR-22-3p) inhibited NLRP3 inflammasome activation and reduced microglia-mediated retinal inflammation in diabetic rats, suggesting that vesicle-based delivery can regulate inflammatory pathways that overlap with PANoptosis-like cell death (169).

Bimetallic oxide nanoparticles, such as Bi_2_Sn_2_O_7_ nanozymes, have been reported to induce PANoptosis mainly in tumor models through disruption of ion homeostasis, mitochondrial dysfunction, ROS generation, and release of DAMPs, thereby enhancing antitumor immune responses (170). However, these findings are primarily derived from non-DR and non-ocular models. Therefore, in the context of DR, Bi_2_Sn_2_O_7_-related nanozyme strategies should be described as hypothesis-generating approaches rather than direct evidence that such nanoparticles can selectively eliminate hyperproliferative neovascular endothelial cells while preserving neuroretinal tissue (171).

Nanomedicine may also facilitate the co-delivery of anti-angiogenic agents and cell-death-modulating compounds. Bevacizumab-loaded nanoparticles have been shown to preserve antiangiogenic activity and improve drug stability, and organic nanocarriers have been discussed as delivery platforms for bevacizumab. In parallel, polymeric systems delivering Necrostatin-1 have shown ocular neuroprotective potential in glaucoma-related models by targeting cell membranes and reducing oxidative stress (172–174). These studies support the feasibility of ocular nanodelivery for anti-VEGF agents or necroptosis-related inhibitors. However, direct evidence that nanoparticles co-encapsulating bevacizumab and Necrostatin-1 can simultaneously suppress neovascularization and PANoptosis in DR remains insufficient. Future studies should determine whether such co-delivery systems modulate bona fide PANoptosome assembly or mainly reduce the co-activation of apoptosis-, pyroptosis-, and necroptosis-related markers in DR-specific models.

### 6.4 Cell-based therapies

MSC-derived exosomes have emerged as promising vehicles for immunomodulatory and neuroprotective therapy in retinal diseases, but their direct use as anti-PANoptotic therapy in DR remains insufficiently validated (168). These nano-sized vesicles can carry bioactive molecules, including miRNAs, lncRNAs, proteins, and lipids, and may regulate inflammation, oxidative stress, apoptosis, and retinal cell survival; however, the specific cargo, recipient cell type, and disease model should be clearly distinguished before extrapolating these findings to DR.

In retinal ischemia/reperfusion injury, TNF-α-stimulated gingival MSC-derived exosomes were tested in mouse retinal ischemia/reperfusion models and oxygen-glucose deprivation/reoxygenation-treated retinal cells, where they reduced neuroinflammation and retinal cell loss through the maternally expressed gene 3 (MEG3)/microRNA-21a-5p (miR-21a-5p) axis rather than through a directly validated miR-124-3p/PANoptosis pathway (175–177). Therefore, MSC-exosome-based delivery in retinal injury supports the feasibility of cell-free neuroprotective therapy, but it should not be described as confirmed anti-PANoptotic therapy in DR unless validated in diabetic retinal models. Similarly, miR-124-3p has been implicated in exosome-mediated neuroimmune regulation and retinal exosome biology, but current evidence does not establish MSC-exosomal miR-124-3p as a DR-specific intervention that directly suppresses PANoptosome assembly. Thus, MSC-derived exosomes should be discussed as exploratory cell-free delivery platforms that may modulate PANoptosis-related inflammatory pathways, rather than as established anti-PANoptotic treatment for DR.

Blocking the stimulator of interferon genes (STING) pathway represents another potential strategy to regulate DNA-sensing-related inflammatory cell death. However, the relationship between STING inhibition and PANoptosis has been demonstrated mainly in non-ocular inflammatory models rather than in DR-specific retinal models. For example, in sepsis-induced acute lung injury models, ursodeoxycholic acid suppressed STING-related PANoptosis and reduced inflammatory lung injury, supporting a mechanistic link between STING signaling and PANoptosis in systemic inflammatory disease (178). Blocking the STING pathway represents another potential strategy to regulate DNA-sensing-related inflammatory cell death. However, the relationship between STING inhibition and PANoptosis has been demonstrated mainly in non-ocular inflammatory models rather than in DR-specific retinal models. For example, in sepsis-induced acute lung injury models, ursodeoxycholic acid suppressed STING-related PANoptosis and reduced inflammatory lung injury, supporting a mechanistic link between STING signaling and PANoptosis in systemic inflammatory disease.

In neuroinflammatory models, the cyclic GMP-AMP synthase (cGAS)-STING pathway has also been linked to mtDNA release, NLRP3 inflammasome activation, and neuronal inflammatory injury. In a mouse model of sevoflurane-induced postoperative cognitive dysfunction, the cGAS inhibitor RU.521 attenuated cognitive dysfunction and NLRP3 inflammasome activation, supporting the role of the mtDNA-cGAS-STING axis in neuroinflammation (179, 180). However, this postoperative cognitive dysfunction model is not a diabetic retinal model, and it does not demonstrate BRB protection or PANoptosome inhibition in DR. Therefore, in the context of DR, STING should be described as a potential DNA-sensing inflammatory target that requires validation in high-glucose retinal cells, STZ-induced diabetic rodents, db/db mice, or human PDR samples. Future studies should determine whether STING inhibition reduces bona fide PANoptosome assembly or only decreases parallel activation of apoptosis-, pyroptosis-, and necroptosis-related markers in diabetic retinal tissues.

### 6.5 Immunomodulation and anti-inflammatory therapy

Immunomodulatory strategies and anti-inflammatory therapies may provide potential approaches for regulating PANoptosis-related inflammatory cell death in DR, but their therapeutic relevance should be interpreted according to the specific immune model in which they were tested. Activated CD4+ T cells and microglia promote neuroinflammation in DR by secreting pro-inflammatory cytokines, which subsequently amplify PANoptosis-related inflammatory signaling via JAK/STAT1 and NF-κB pathways (181). However, direct evidence that CD4+ T-cell or IFN-γ blockade suppresses bona fide PANoptosome assembly in diabetic retinal tissues remains limited. In a non-diabetic mouse retinal ischemia/reperfusion model, CD4+ T-cell infiltration and microglial activation accompanied progressive RGC loss, and T-cell deficiency or IFN-γ neutralization attenuated retinal neurodegeneration and functional impairment (110, 111). In a diabetic mouse corneal nerve degeneration model, CD4+CD25− T cells secreted IFN-γ, and IFN-γ blockade alleviated trigeminal ganglion neurite inhibition and corneal nerve degeneration; however, this model reflects diabetic ocular neuropathy rather than DR-specific retinal PANoptosis (111). Therefore, CD4+ T cell/IFN-γ-mediated immune crosstalk should be interpre ted as a mechanism relevant to ocular neuroinflammatory injury under ischemic or diabetic conditions, but its therapeutic value in DR still requires validation in STZ-induced diabetic rodents, db/db mice, high-glucose retinal cells, or human DR/PDR retinal samples.

Immune checkpoint biology may provide conceptual insight into T-cell-mediated immune regulation, but systemic immune checkpoint blockade should not currently be presented as a plausible therapeutic strategy for DR. Unlike local anti-inflammatory modulation, programmed cell death protein 1 (PD-1)/programmed death-ligand 1 (PD-L1) or cytotoxic T-lymphocyte-associated protein 4 (CTLA-4) blockade can produce broad immune activation and immune-related adverse events. Importantly, immune checkpoint inhibitor-induced diabetes mellitus has been reported in patients receiving cancer immunotherapy, with a large cohort study reporting a prevalence of 0.45% among immune checkpoint inhibitor (ICI)-treated patients and other reviews reporting an incidence of approximately 1–2%; this complication may present acutely and can progress to diabetic ketoacidosis (146, 182). Given that DR occurs in patients with pre-existing diabetes, the metabolic and autoimmune risks of systemic checkpoint inhibition are particularly relevant. Therefore, in the context of DR, immune checkpoint pathways should be discussed mainly as cautionary examples of systemic immunomodulation rather than as candidate therapeutic targets. Future immunomodulatory strategies should prioritize local, pathway-specific, and retina-targeted approaches with careful metabolic safety monitoring.

Collectively, these considerations underscore the multifaceted role of immunity in PANoptosis regulation while also highlighting the risks of broad systemic immunomodulation. Translating these findings into DR therapy requires a careful balance between suppressing pathological inflammation and preserving host defense, metabolic stability, and tissue repair. At present, locally delivered, pathway-specific anti-inflammatory strategies and ncRNA-based approaches appear more appropriate for further preclinical evaluation than systemic immune checkpoint blockade.

## 7 Challenges and future directions

### 7.1 Translational medicine bottlenecks

The translation of PANoptosis-targeted therapies for DR faces significant challenges, primarily due to the unique anatomical and physiological barriers of the eye. The BRB, a selective permeability barrier, restricts systemic drug delivery to retinal tissues. Current nanomedicine approaches, though promising, require optimization of particle size (ideally <100 nm) to enhance trans-barrier penetration while avoiding inflammation. For instance, pH-responsive poly(lactic-co-glycolic acid) (PLGA) nanoparticles loaded with caspase inhibitors demonstrate improved retinal accumulation in preclinical models, but their clinical translation is impeded by inconsistent drug loading efficiency and potential retinal toxicity.

Specificity continues to pose a significant challenge. PANoptosis pathways share components with fundamental immune surveillance mechanisms, including caspase-8 and RIPK3, whose systemic inhibition could increase susceptibility to infections. Preclinical studies employing pan-caspase inhibitors have reported increased bacterial clearance defects in animal models, highlighting the necessity for cell-type-specific interventions. The development of neuron- or endothelial-specific inhibitors, such as viral vector-driven caspase-3 Short hairpin RNA (shRNA) targeting RGCs, may reduce off-target effects while maintaining systemic immunity.

Clinical validation of PANoptosis biomarkers presents another challenge. Although the six-gene signature (BEX2, CASP2, CD36, FASN, OSMR, PLSCR3) demonstrates diagnostic utility in small cohorts, large-scale longitudinal studies are required to confirm their prognostic value and to guide patient stratification. Moreover, most preclinical models depend on rodent DR models, which inadequately replicate the complexity of the human retina, thus necessitating the development of humanized organoids or non-human primate models for translational research.

### 7.2 Unresolved scientific questions

The field of PANoptosis in DR faces critical, unresolved questions that must be addressed to advance our mechanistic understanding and therapeutic development. One notable gap is the role of sex-based differences in PANoptosis regulation. While preclinical studies in other inflammatory diseases suggest that estrogen may modulate ZBP1 expression via estrogen receptor α (ERα), its impact on PANoptosis in DR remains unexplored. Translating this to DR, sex-specific differences in PANoptosis may underlie gender disparities in DR prevalence and progression, warranting studies to validate the estrogen-ERα-ZBP1 axis in retinal cells.

An unresolved challenge is defining the reversibility of PANoptosis in early DR. While acute retinal ischemia/reperfusion models indicate that PANoptosis inhibitors can reduce neuronal loss when administered within 24 hours of injury, the therapeutic window for intervening in DR’s chronic hyperglycemia-driven PANoptosis is not yet clear. Key questions include: 1) Can early-stage PANoptosis in the retinal microvasculature be halted without disrupting physiological cell turnover? 2) How do neuroprotective mechanisms differ from pro-reparative responses in PANoptosis inhibition? Preclinical data in glaucoma suggest that delaying PANoptosis inhibition beyond inflammation resolution may impair tissue repair, emphasizing the need to distinguish between acute anti-inflammatory and chronic reparative phases. Moreover, the heterogeneity of PANoptosis pathways across different DR subtypes, such as non-proliferative versus proliferative DR, has yet to be fully characterized. It remains to be confirmed whether these cellular subsets respond differently to therapeutic interventions. Comprehending this heterogeneity will be crucial for the development of subtype-specific treatments.

In summary, addressing sex-specific mechanisms, defining therapeutic windows for reversibility, and characterizing the heterogeneity of PANoptosis are essential for translating basic discoveries into precision treatments for DR.

### 7.3 Context-dependent risks and safety considerations of PANoptosis modulation

Although excessive PANoptosis-related inflammatory cell death may aggravate neurovascular injury in DR, complete or systemic inhibition of PANoptosis is unlikely to be uniformly beneficial. PANoptosis-related molecules, including caspase-8, RIPK1/RIPK3, NLRP3, AIM2, and ZBP1, are not disease-specific mediators; they also participate in host defense, innate immune surveillance, cytokine regulation, and clearance of infected or damaged cells (8, 11).

Therefore, indiscriminate blockade of these pathways may impair antimicrobial responses, disturb physiological inflammatory resolution, or interfere with retinal repair after injury (104). This is particularly relevant for DR, which is a chronic and heterogeneous disease involving early neurodegeneration, vascular leakage, ischemia, diabetic macular edema, and proliferative neovascularization.

The biological consequences of PANoptosis modulation may also vary across disease stages and retinal cell types. In early DR, limited suppression of excessive inflammatory cell death in RGCs, Müller cells, microglia, or endothelial cells may be neuroprotective or vasculoprotective. In advanced proliferative disease, however, the same intervention may have different effects on ischemia-driven angiogenesis, fibrovascular remodeling, immune-cell infiltration, and repair-associated inflammation.

These considerations suggest that PANoptosis should be viewed as a therapeutic window rather than a simple on/off target. Future strategies should prioritize biomarker-guided patient selection, local intraocular delivery, reversible or temporally controlled modulation, and retinal cell-type-specific validation before clinical translation.

### 7.4 Frontier research directions

Frontier technologies, including artificial intelligence (AI)-assisted modeling, retinal organoids, liquid biopsy, genome editing, and combination therapy, may provide useful platforms for studying PANoptosis-related mechanisms in DR. However, their clinical value remains to be established through DR-specific validation, and these approaches should be interpreted as exploratory research directions rather than near-term clinical strategies.

AI-assisted modeling may help prioritize PANoptosis-related molecular networks, candidate biomarkers, and potential therapeutic targets by integrating transcriptomic, imaging, and clinical datasets. Machine learning models based on DR-related molecular signatures may improve patient stratification after external validation using large-scale, longitudinal, and multicenter datasets. However, AI-derived candidates should not be considered therapeutic targets solely on the basis of computational prediction; functional validation in high-glucose retinal cells, diabetic animal models, and human DR/PDR samples remains necessary.

Retinal organoids may help model cell-type-specific responses under hyperglycemic or inflammatory stress, particularly in retinal neurons, Müller cells, RPE cells, and vascular-like cell populations. Nevertheless, current retinal organoid systems cannot yet fully recapitulate the vascular, immune, metabolic, and blood–retinal barrier complexity of human DR. Therefore, organoid-based studies should be regarded as complementary platforms for mechanistic exploration and drug screening, rather than definitive models that directly visualize or confirm PANoptosis in human diabetic retina.

Liquid biopsy approaches may provide candidate noninvasive biomarkers for DR progression and treatment response. Exosomal cargoes such as inflammatory cell death-related proteins, cytokines, miRNAs, or mtDNA fragments may reflect retinal stress or systemic inflammatory activity. However, their ability to indicate active retinal PANoptosis in DR requires specimen-specific and longitudinal validation using serum, plasma, aqueous humor, vitreous fluid, and matched clinical phenotypes. Therefore, liquid biopsy markers should be described as candidate signals rather than confirmed readouts of retinal PANoptosome activation.

Genome editing technologies, including clustered regularly interspaced short palindromic repeats and CRISPR-associated protein 9 (CRISPR-Cas9) and AAV-based delivery systems, may provide tools for mechanistic studies of PANoptosis-related molecules such as ZBP1, NLRP3, RIPK3, or caspase-8. However, direct evidence supporting therapeutic CRISPR-mediated suppression of PANoptosis in diabetic retinal tissues remains limited. Before clinical translation, these approaches require rigorous validation of retinal cell-type specificity, delivery efficiency, off-target effects, immune safety, and long-term consequences of modulating molecules that also participate in host defense and tissue repair.

Combination strategies integrating PANoptosis-related modulation with established DR treatments, such as anti-VEGF therapy, may represent a future direction for multifactorial intervention. However, current evidence is insufficient to conclude that RIPK3 inhibition and anti-VEGF therapy synergistically suppress PANoptosis and vascular leakage in DR. Future studies should determine whether such combinations provide additive or synergistic benefits in diabetic animal models and whether they modulate bona fide PANoptosome assembly or mainly reduce apoptosis-, pyroptosis-, and necroptosis-related marker co-activation.

Overall, frontier technologies may support future precision research in DR by improving mechanistic modeling, biomarker discovery, retinal cell-type-specific validation, and therapeutic screening. Nevertheless, these approaches should be developed within a cautious evidence-based framework. DR-specific validation, longitudinal biomarker cohorts, standardized model systems, and safety evaluation will be essential before PANoptosis-related frontier technologies can be translated into clinical decision-making.

Frontier technologies, including AI-assisted modeling, retinal organoids, liquid biopsy, genome editing, and combination therapy, may provide useful platforms for studying PANoptosis-related mechanisms in DR. However, their clinical value remains to be established through DR-specific validation. AI-based prognostic models may improve patient stratification only after external validation using longitudinal clinical, imaging, and molecular datasets. Retinal organoids may help model cell-type-specific responses under hyperglycemic or inflammatory stress, but they cannot yet fully recapitulate the vascular, immune, metabolic, and blood–retinal barrier complexity of human DR. Similarly, liquid biopsy and CRISPR-based approaches should be regarded as exploratory tools that require rigorous validation of sensitivity, specificity, specimen source, delivery safety, off-target effects, and long-term biological consequences before clinical application. Therefore, these technologies may support future precision research in DR, but they should not yet be interpreted as established diagnostic or therapeutic strategies for PANoptosis-targeted clinical management.

## 8 Conclusion

PANoptosis, a coordinated inflammatory cell death program involving apoptosis, pyroptosis, and necroptosis, provides a useful but still evolving framework for interpreting inflammatory neurovascular injury in DR. Current evidence suggests that PANoptosis-related pathways, including MAPK/ROS signaling, TNF-α-associated inflammatory signaling, ncRNA-mediated regulation, metabolic stress, and immune-cell crosstalk, may contribute to retinal inflammation, endothelial dysfunction, and neuronal loss. However, the extent to which complete PANoptosome assembly occurs across different stages of DR and across distinct retinal cell types remains incompletely defined. Therefore, PANoptosis should be interpreted as an emerging mechanistic concept rather than a fully established central driver of DR pathology.

From a translational perspective, targeting PANoptosis-related pathways may complement existing vascular-, inflammatory-, and metabolism-centered therapeutic strategies, particularly when interventions are locally delivered, temporally controlled, and guided by validated biomarkers. Nevertheless, broad or systemic inhibition of PANoptosis may not be uniformly beneficial, because several key PANoptosis-related molecules, including caspase-8, RIPK1/RIPK3, NLRP3, AIM2, and ZBP1, also participate in host defense, innate immune surveillance, cytokine regulation, and the clearance of infected or damaged cells. Thus, future therapeutic development should emphasize biomarker-guided patient selection, retinal cell-type-specific validation, localized intraocular delivery, and careful evaluation of immune and reparative risks.

Future studies should prioritize direct DR-specific validation of PANoptosome assembly, longitudinal evaluation of candidate biomarkers, and mechanistic analyses across different retinal cell types and disease stages. The integration of multi-omics data, retinal imaging, retinal organoid models, and carefully designed multicenter studies may help determine whether PANoptosis-related pathways can be translated into clinically useful tools for DR risk stratification and therapeutic decision-making. In this context, PANoptosis is best viewed not as a universally beneficial inhibition target, but as a context-dependent inflammatory cell death framework that requires rigorous validation before clinical translation.

## Glossary

DR: diabetic retinopathy
VEGF: vascular endothelial growth factor
PCD: programmed cell death
RIPK3: Receptor-interacting serine-threonine kinase 3
NLRP3: NOD-like receptor family pyrin domain-containing 3
IL-1β: interleukin-1 beta
DAMPs: danger-associated molecular patterns
Drp1: dynamin-related protein 1
ROS: reactive oxygen species
mtDNA: mitochondrial DNA
ncRNAs: non-coding RNAs
NADPH: nicotinamide adenine dinucleotide phosphate
BRB: blood-retinal barrier
AGEs: advanced glycation end products
TNF-α: tumor necrosis factor alpha
IL-6: interleukin-6
RGCs: retinal ganglion cells
NETs: neutrophil extracellular traps
AIM2: absent in melanoma 2
CCL2: C-C motif chemokine ligand 2
MCP-1: monocyte chemoattractant protein-1
IL-17A: interleukin-17A
CXCL1: C-X-C motif chemokine ligand 1
CXCL8: C-X-C motif chemokine ligand 8
ICAM-1: intercellular adhesion molecule 1
VCAM-1: vascular cell adhesion molecule 1
IL-18: interleukin-18
PDR: proliferative diabetic retinopathy
PKC: protein kinase C
MAPK: mitogen-activated protein kinase
PEDF: pigment epithelium-derived factor
MMP2/MMP9: matrix metalloproteinase
NMDA: N-methyl-D-aspartic acid
M1: classically activated pro-inflammatory macrophage/microglial phenotype
NO: nitric oxide
BDNF: brain-derived neurotrophic factor
TrkB: tropomyosin receptor kinase B
AKT: protein kinase B
ERK: extracellular signal-regulated kinase
NGF: nerve growth factor
TrkA: tropomyosin receptor kinase A
CNTF: ciliary neurotrophic factor
GDNF: glial cell line-derived neurotrophic factor
STZ: streptozotocin
VEGFR2: vascular endothelial growth factor receptor 2
RAGE: receptor for advanced glycation end products
NF-κB: nuclear factor kappa-light-chain-enhancer of activated B cells
FFAs: free fatty acids
TLR4: toll-like receptor 4
MyD88: myeloid differentiation primary response 88
4-HNE: 4-hydroxynonenal
MDA: malondialdehyde
ALEs: advanced lipoxidation end products
FDP-Lysine: Nϵ-(3-formyl-3, 4-dehydropiperidino)lysine
miR-34a: microRNA-34a
p53: tumor protein p53
SIRT1: sirtuin 1
HECTD1-AS1: HECT domain E3 ubiquitin protein ligase 1 antisense RNA 1
miR-124-3p: microRNA-124-3p
RPE: retinal pigment epithelium
ZBP1: Z-DNA-binding protein 1
Mdivi-1: mitochondrial division inhibitor 1
p-MLKL: phosphorylated mixed lineage kinase domain-like protein
IFN-γ: interferon-gamma
JAK: Janus kinase
STAT1: signal transducer and activator of transcription 1
IRF1: interferon regulatory factor 1
iNOS: inducible nitric oxide synthase
Fas: Fas cell surface death receptor
FADD: Fas-associated death domain protein
MLKL: mixed lineage kinase domain-like protein
miRNAs: microRNAs
lncRNAs: long non-coding RNAs
circRNAs: circular RNAs
miR-181a: microRNA-181a
KCNQ1OT1: KCNQ1 overlapping transcript 1
miR-17-5p: microRNA-17-5p
TXNIP: thioredoxin-interacting protein
AQP4-AS1: aquaporin 4 antisense RNA 1
CDR1as: cerebellar degeneration-related protein 1 antisense RNA
miR-7: microRNA-7
dsDNA: double-stranded DNA
miR-423-5p: microRNA-423-5p
NOD2: nucleotide-binding oligomerization domain-containing protein 2
CD4+: T cells cluster of differentiation 4-positive T cells
TNFR1: tumor necrosis factor receptor 1
DKK1: Dickkopf-1
GSDMD: gasdermin D
ASC: apoptosis-associated speck-like protein containing a CARD
NLRP12: NOD-like receptor family pyrin domain-containing 12
HSV-1: herpes simplex virus 1
ELISA: enzyme-linked immunosorbent assay
FUNDC1: FUN14 domain containing protein 1
O-GlcNAc: O-linked N-acetylglucosamine
PPARγ: peroxisome proliferator-activated receptor gamma
RXRα: retinoid X receptor alpha
Wnt: wingless-type MMTV integration site family
LRP5/6: low-density lipoprotein receptor-related protein 5/6
BEX2: brain expressed X-linked 2
CASP2: caspase-2
CD36: cluster of differentiation 36
FASN: fatty acid synthase
OSMR: oncostatin M receptor
PLSCR3: phospholipid scramblase 3
scRNA-seq: single-cell RNA sequencing
Aβ1–40: amyloid-beta peptide 1-40
AMD: age-related macular degeneration
PYRIN: pyrin inflammasome sensor
tMCAO: transient middle cerebral artery occlusion
OGD/Re: oxygen-glucose deprivation/reoxygenation
MSCs: mesenchymal stem cells
mtROS: mitochondrial reactive oxygen species
GSDMD-N: N-terminal fragment of gasdermin D
LC3: microtubule-associated protein 1 light chain 3
ATG12: autophagy-related protein 12
RNAi: RNA interference
siRNA: small interfering RNA
HUVECs: human umbilical vein endothelial cells
AAV: adeno-associated virus
NEDD4: neural precursor cell expressed developmentally downregulated 4
PTEN: phosphatase and tensin homolog
NRF2: nuclear factor erythroid 2-related factor 2
HIF-1α: hypoxia-inducible factor 1 alpha
EZH2: enhancer of zeste homolog 2
PGC-1α: peroxisome proliferator-activated receptor gamma coactivator 1-alpha
miR-22-3p: microRNA-22-3p
MEG3: maternally expressed gene 3
miR-21a-5p: microRNA-21a-5p
STING: stimulator of interferon genes
cGAS: cyclic GMP-AMP synthase
PD-1: programmed cell death protein 1
PD-L1: programmed death-ligand 1
CTLA-4: cytotoxic T-lymphocyte-associated protein 4
ICI: immune checkpoint inhibitor
PLGA: poly(lactic-co-glycolic acid)
shRNA: short hairpin RNA
ERα: estrogen receptor alpha
AI: artificial intelligence
CRISPR-Cas9: clustered regularly interspaced short palindromic repeats and CRISPR-associated protein 9
